# Supplementary material for: Repeated evolution of asymmetric genitalia and right-sided mating behavior in the Drosophila nannoptera species group
Source: BMC Evol Biol. 2019 May 27;19:109. doi: 10.1186/s12862-019-1434-z (PMC6537454; doi:10.1186/s12862-019-1434-z)
Supplement: Supplementary file 1 — Supplementary Figures and Tables. Figure S1 The aedeagus of D. pachea is asymmetric. Figure S2. The aedeagus of D. acanthoptera is asymmetric. Figure S3. No asymmetry is detected in the aedeagus of D. nannoptera. Figure S4. No asymmetry is detected in the aedeagus of D. machalilla. Figure S5. No asymmetry is detected in the aedeagus of D. bromeliae. Figure S6. Courtship and copulation duration in D. pachea and related species. Figure S7. Multi-species mating position measurements. Figure S8. D. nannoptera tilts to the right side of the female abdomen. Table S1 Species Resources, Table S2 GenBank Accession Numbers of the phylogeny dataset. (PDF 2543 kb) [file 12862_2019_1434_MOESM1_ESM.pdf]

Table S1: Species Resources

Species	Source	Stock number	collection locality	collection year
D. acanthoptera	Drosophila Species Stock Center	15090-1693.00	Oaxaca, Mexico	1976
D. pachea	Drosophila Species Stock Center	15090-1698.02	Sonora, Mexico	1996
D. nannoptera	Drosophila Species Stock Center	15090-1692.10<br>15090-1698.12	Oaxaca/Puebla, Mexico	1992
D. machalilla	Andrea Acurio		San Jose, Ecuador	2015
D. bromeliae	Drosophila Species Stock Center	15085-1682.00	Grand Cayman Island, UK	1985
D. buzzatii	Jean David		Bahia, Brazil	2010
D. mojavensis	Drosophila Species Stock Center	15081-1352.22	Catalina Island, USA	2002
D. tripunctata	Drosophila Species Stock Center	15020-2401,02	New Orleans, USA	1950
D. willistoni	Jean David		Rio de Janeiro, Brazil	2010
D. melanogaster	Drosophila Species Stock Center	14021-0231.07	Taiwan	1968

Table S2: GenBank Accession Numbers of the phylogeny dataset

	Locus, Accession Number										
**Species**	***amy***	***amyrel***	***boss***	***fkh***	***marf***	***sinA***	***snf***	***wee***	**ND2**	**COI**	**COII**
D. acanthoptera	KF632687	KF632675	JF736442	KF632652	KF632638	EU341611	JF736382	KF632612	KF632701	KF632601	AF183968
D. pachea	KF632697	KF632683	KF632672	KF632662	KF632648	KF632595	KF632634	KF632622	KF632709	KF632609	KF632600
D. nannoptera	KF632696	KF632682	JF736456	KF632661	KF632647	JF736334	KF632633	KF632621	KF632708	DQ471531	AF183971
D. machalilla	KF632694	KF632680	KF632671	KF632659	KF632645	KF632594	KF632631	KF632619	KF632706	KF632607	KF632599
D. bromeliae	KF632689	AY733049	KF632666	KF632654	KF632640	KF632591	KF632627	KF632614	KF632702	KF632602	AF478418
D. buzzatii	KF632690	KF632677	KF632667	KF632655	KF632641	EU341621	JF736384	KF632615	KF632703	KF632603	DQ202011
D. mojavensis	XM002004425	XM002006561	XM001999692	XM001999791	XM002009489	XM002007289	XM002011475	XM002003093	BK006339	BK006339	BK006339
D. tripunctata	---	---	---	---	---	---	---	---	EU493508	EF570023	EU493748
D. willistoni	CH963849	CH963719	CH964272	CH964232	CH963925	CH963876	CH964239	CH963920	consensus of:<br>NW002031144<br>NW002033407<br>NW002033850<br>NW002036038<br>NW002038401	JQ679116	EU532097
D. melanogaster	NM079044	NM057914	NM080709	NM079818	AF355475	NM057377	NM078490	NM057687	NC024511	NC024511	NC024511

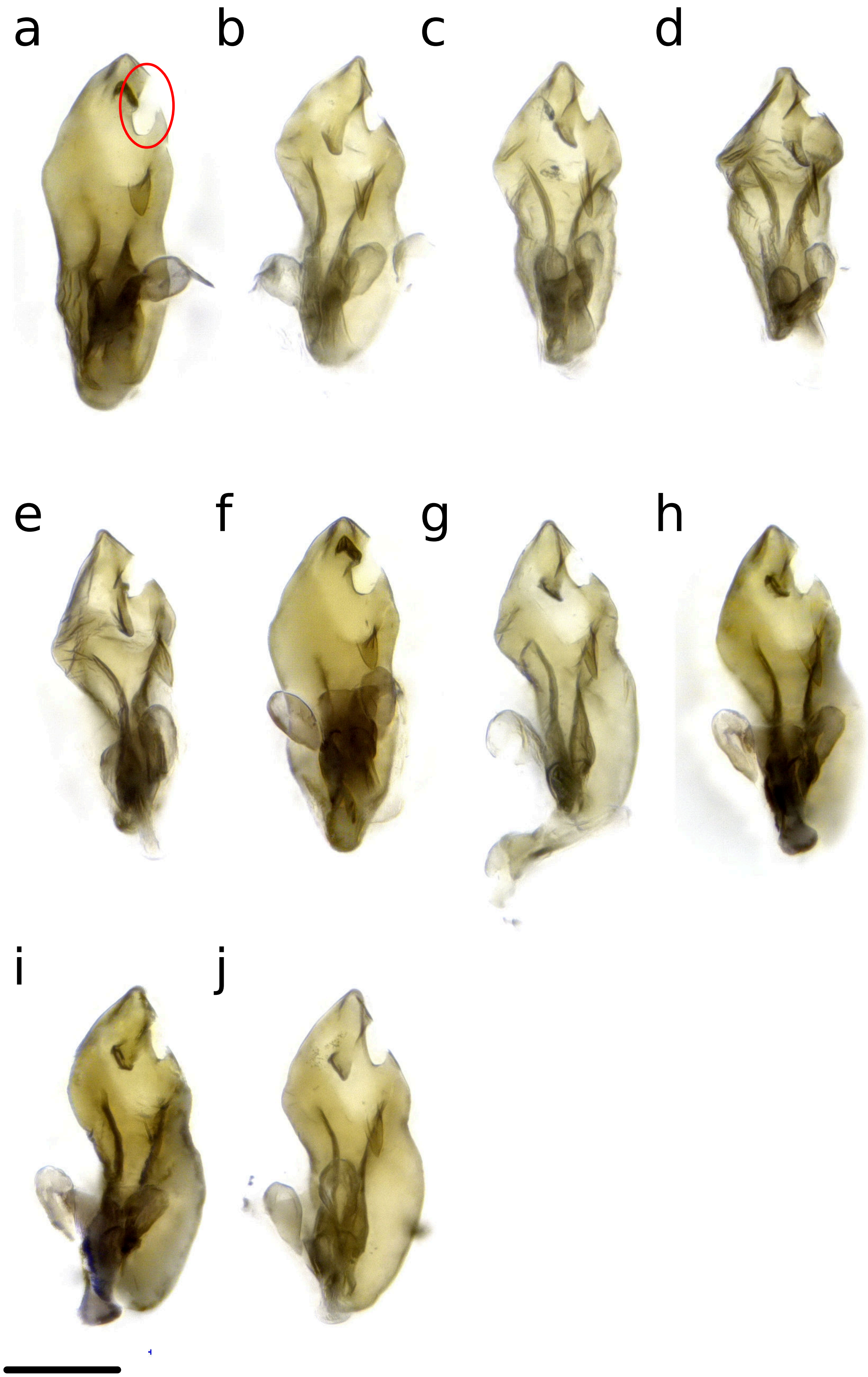

Figure S1: The aedeagus of *D. pachea* is asymmetric. Preparations in ventral view **(a)** The red circle indicates the right-sided position of the gonopore. **(a-j)** Ten preparations. The scale bar is 100 µm.

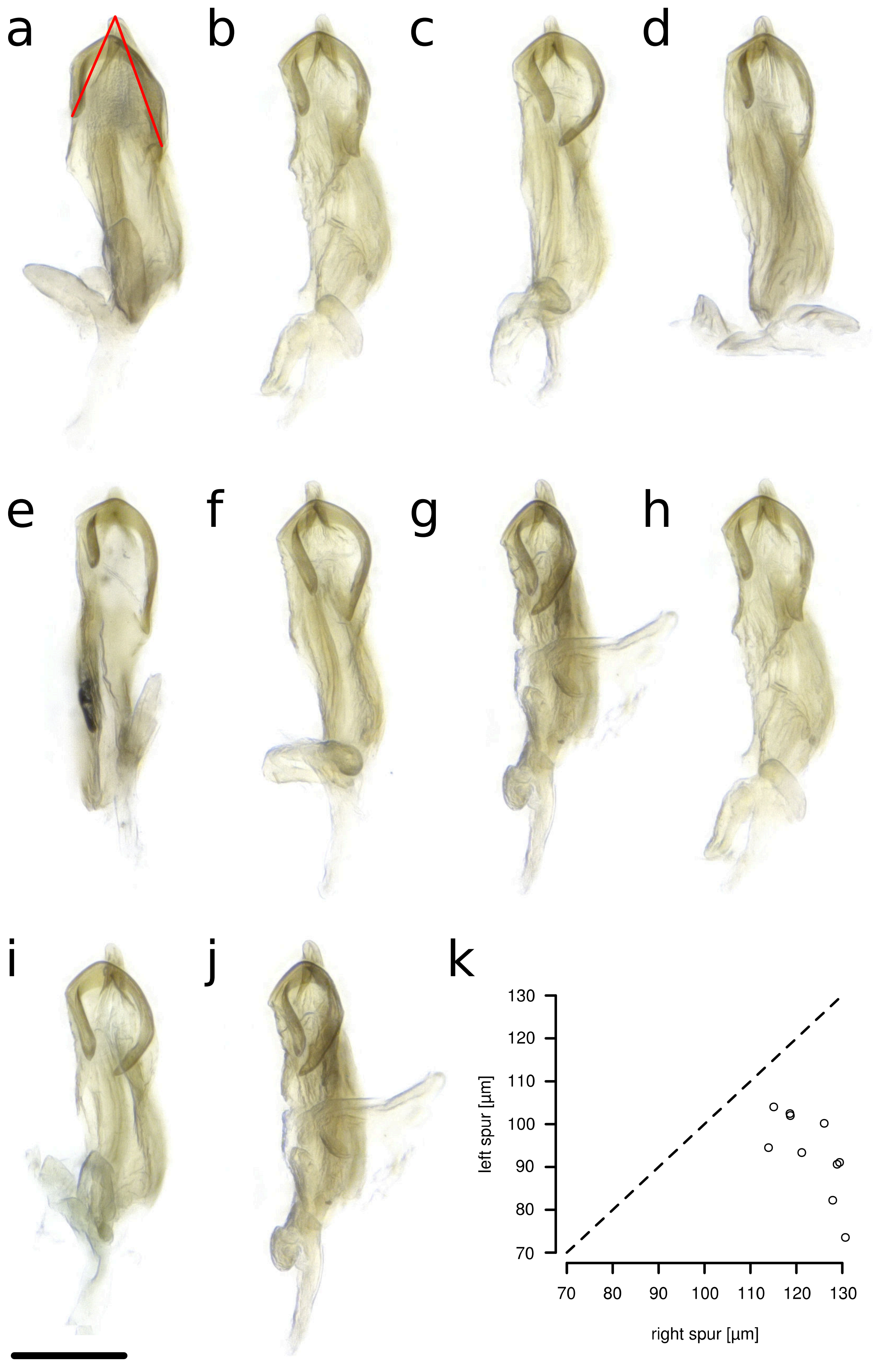

Figure S2: The aedeagus of *D. acanthoptera* is asymmetric. Preparations in ventral view. **(a)** The red lines indicate the length measurements of ventral apex spurs (see materials and methods). **(a-j)** preparations. **(k)** Length measurements of apical spurs. The dashed line corresponds to the 1:1 length ratio of left and right spurs. The scale bar is 100 µm.

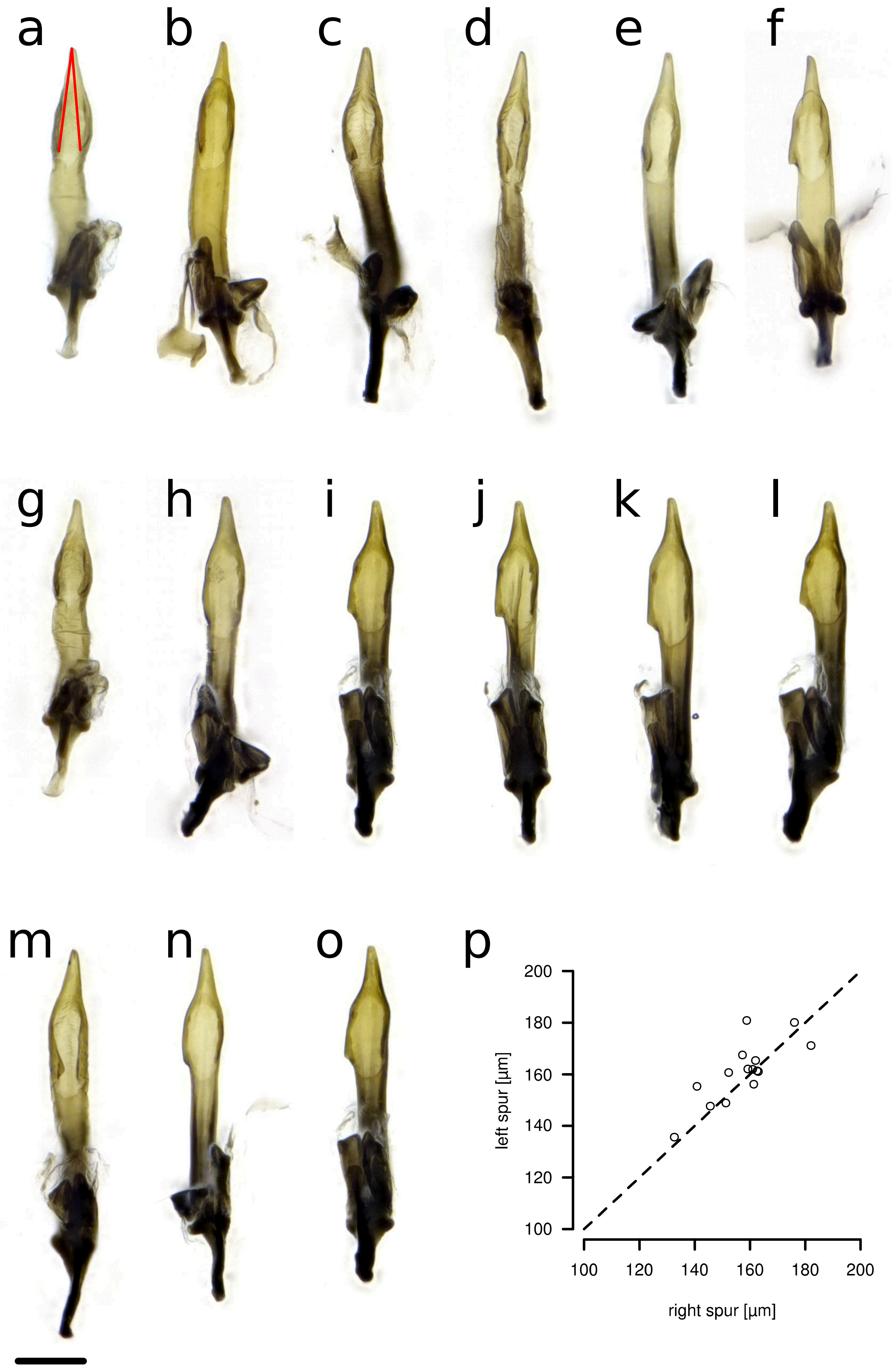

Figure S3: No asymmetry is detected in the aedeagus of *D. nannoptera*. Preparations in ventral view **(a)** The red lines indicate the length measurements of ventral apex spurs (see materials and methods). **(a-o)** Fifteen preparations. **(p)** Length measurements of apical spurs. The dashed line corresponds to the 1:1 length ratio of left and right spurs. The scale bar is 100 µm.

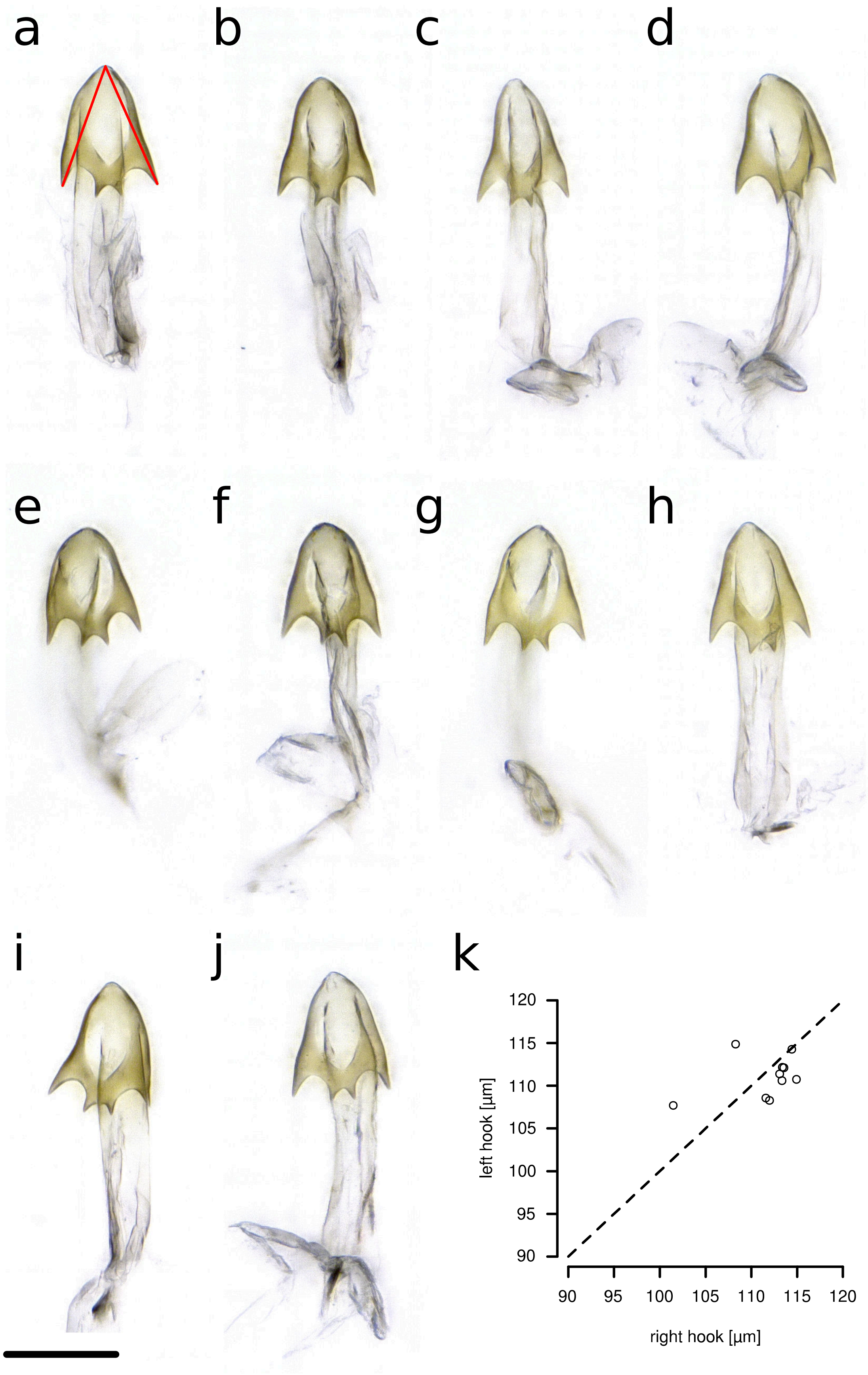

Figure S4: No asymmetry is detected in the aedeagus of *D. machalilla*. Preparations in ventral view **(a)** The red lines indicate the length measurements of ventral apical hooks (see materials and methods). **(a-j)** Ten preparations. **(k)** Length measurements of apical hooks. The dashed line corresponds to the 1:1 length ratio of left and right hooks. The scale bar is 100 µm.

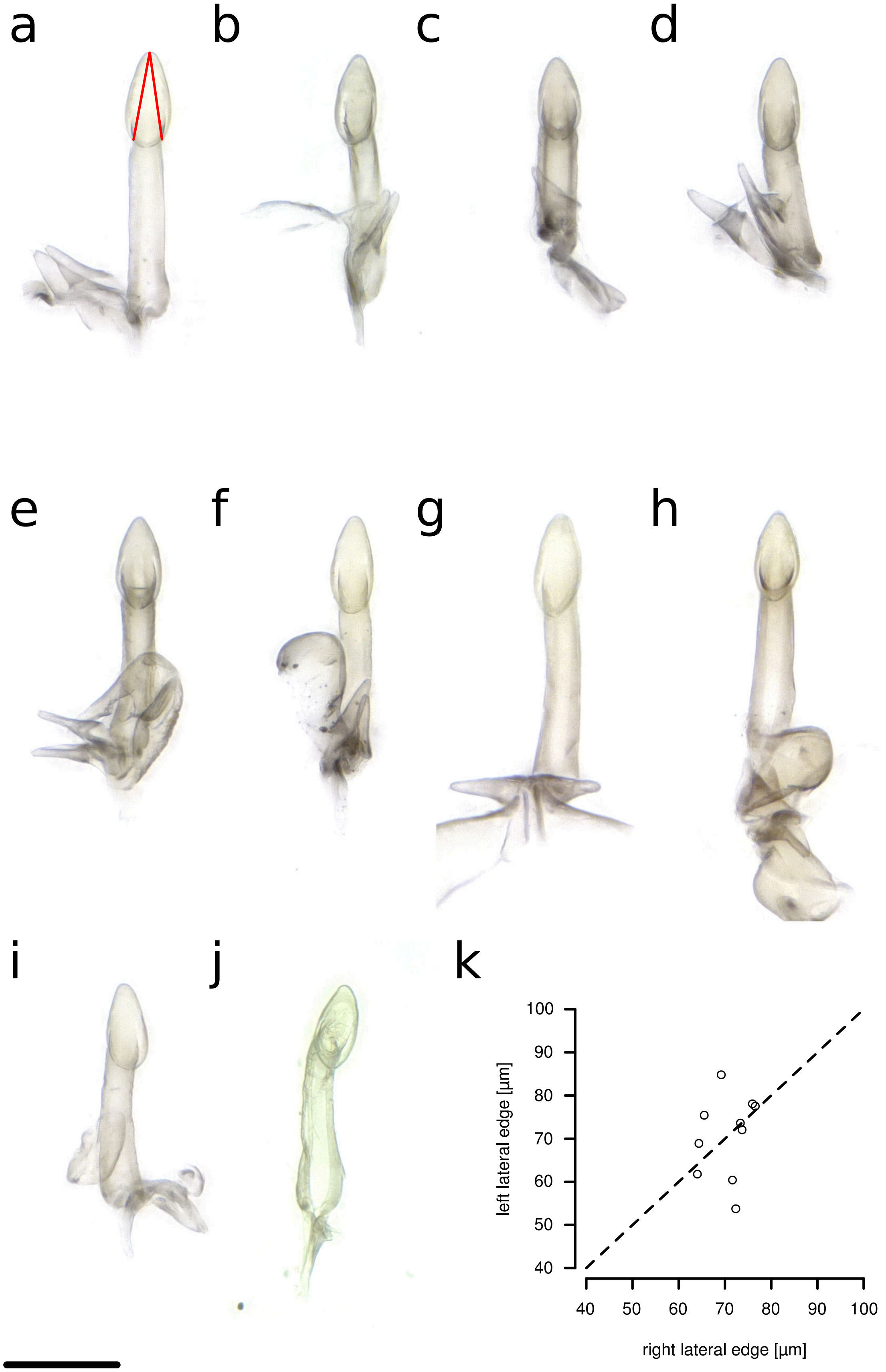

Figure S5: No asymmetry is detected in the aedeagus of *D. bromeliae*. Preparations in ventral view **(a)** The red lines indicate the length measurements of ventral apex ridges (see materials and methods). **(a-j)** Replicate preparations. **(k)** Length measurements of apex ridges. The dashed line corresponds to the 1:1 length ratio of left and right ridges. The scale bar is 100 µm.

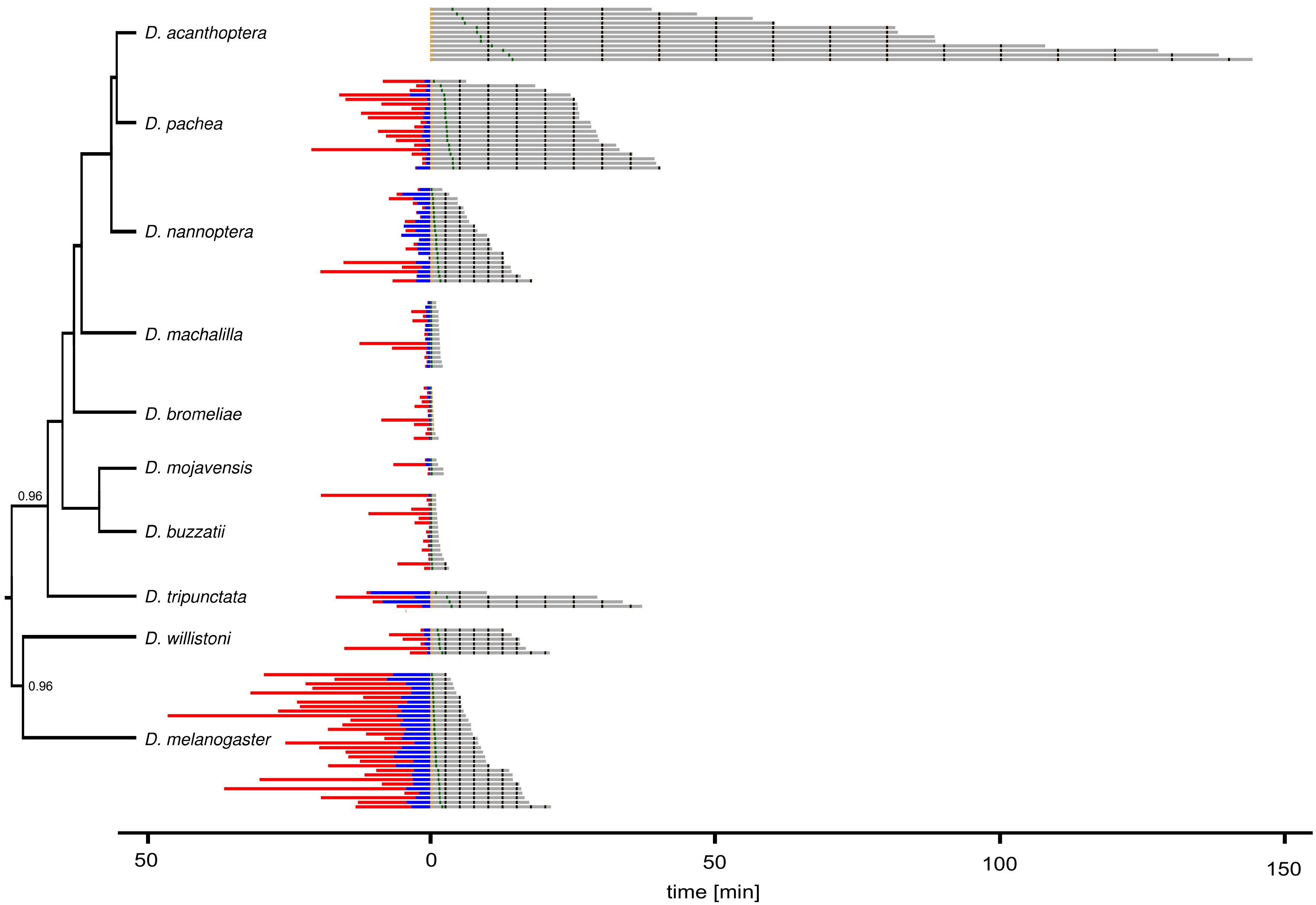

Figure S6: Courtship and copulation duration in *D. pachea* and related species. The phylogenetic relationships of analyzed species are indicated on the left with a bayesian phylogeny based on a multilocus dataset of Lang et al. 2014 [28], and additional data for *D. willistoni* and *D. tripunctata* (see material and methods, Additional file 1: Table S1). Numbers indicate posterior probabilities for node supports < 1. Each line represents an experiment. Courtship is indicated in red, initial copulation with variable positions in blue and copulation after the settling time point in grey. Experiments are aligned by the settling time point. Time points at which the mating angle was calculated are indicated as tick marks: the settling time point in yellow; 10% stable copulation time point in green and measurements at later regular time intervals in black.

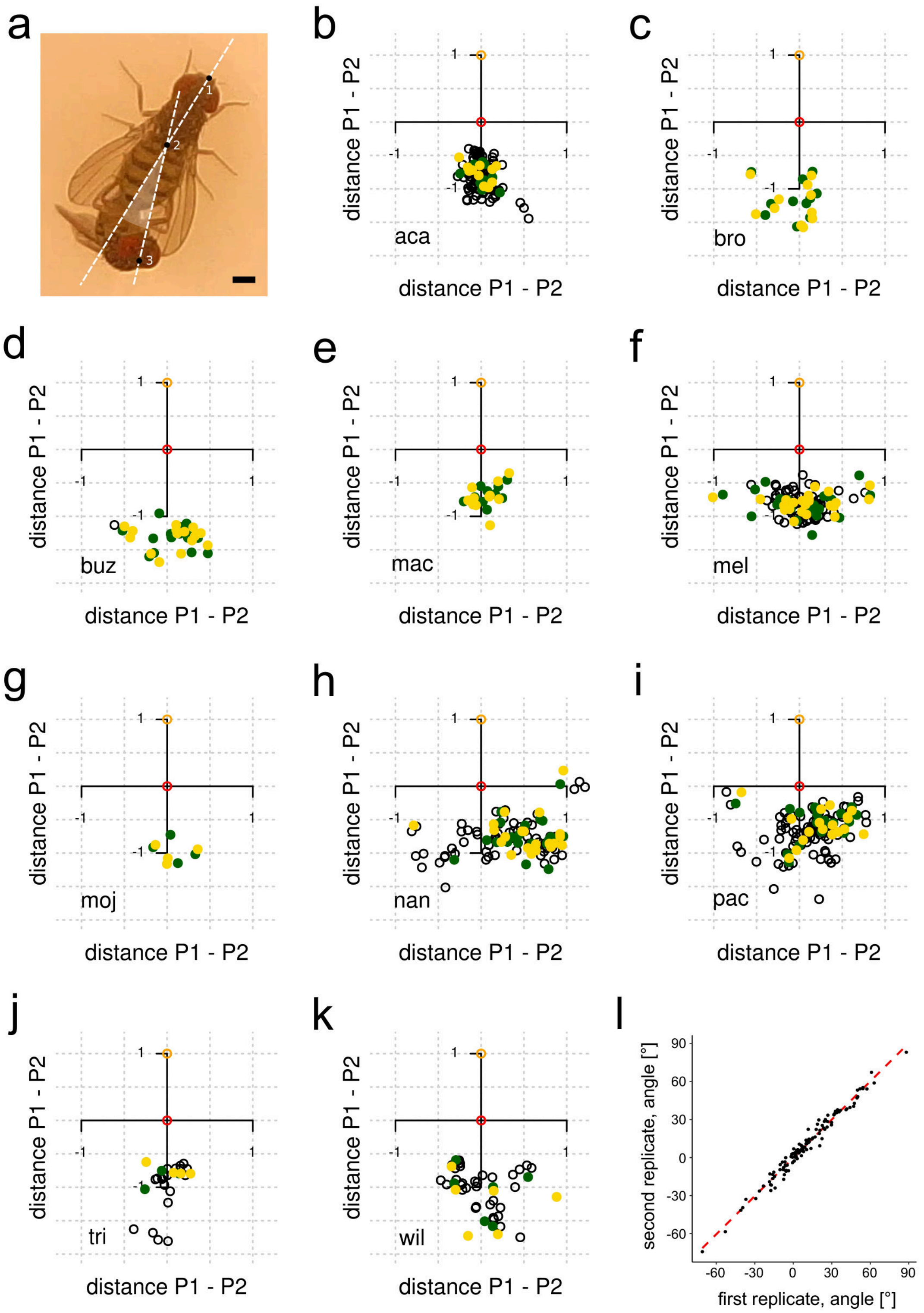

Figure S7: Multi-species mating position measurements. (a) Mating couple of *D. buzzatii* the scale bar 500 µm. Landmarks P1 (1), P2 (2), and P3 (3) are indicated. The dashed white lines (P1,P2) and (P2,P3) form an acute angle (semi-transparent circle sectors) which was measured to assess copulation posture. **(b-k)** Position coordinates for angle measurements of *D. pachea* and nine related *Drosophila* species; aca: *D. acanthoptera*, bro: *D. bromeliae*, buz: *D. buzzatii*, mac: *D. machalilla*, mel: *D. melanogaster*, moj: *D. mojavensis*, nan: *D. nannoptera*, pac: *D. pachea*, tri: *D. tripunctata*, wil: *D. willistoni*. Points P1 (orange circle) are placed at coordinates (0,1) and P2 (red circle) at coordinates (0,0). P3 points are shown for the settling time-point in yellow dots, for the 10% stable copulation time point in green dots and for later time points in black circles. **(l)** Correlation of angle values calculated from two replicate measurements (n=124) at the 10% copulation time point (Pearson correlation coefficient = 0.988, df = 121, t = 69.231, p < 10e -16). The red dashed line indicates the linear regression line.

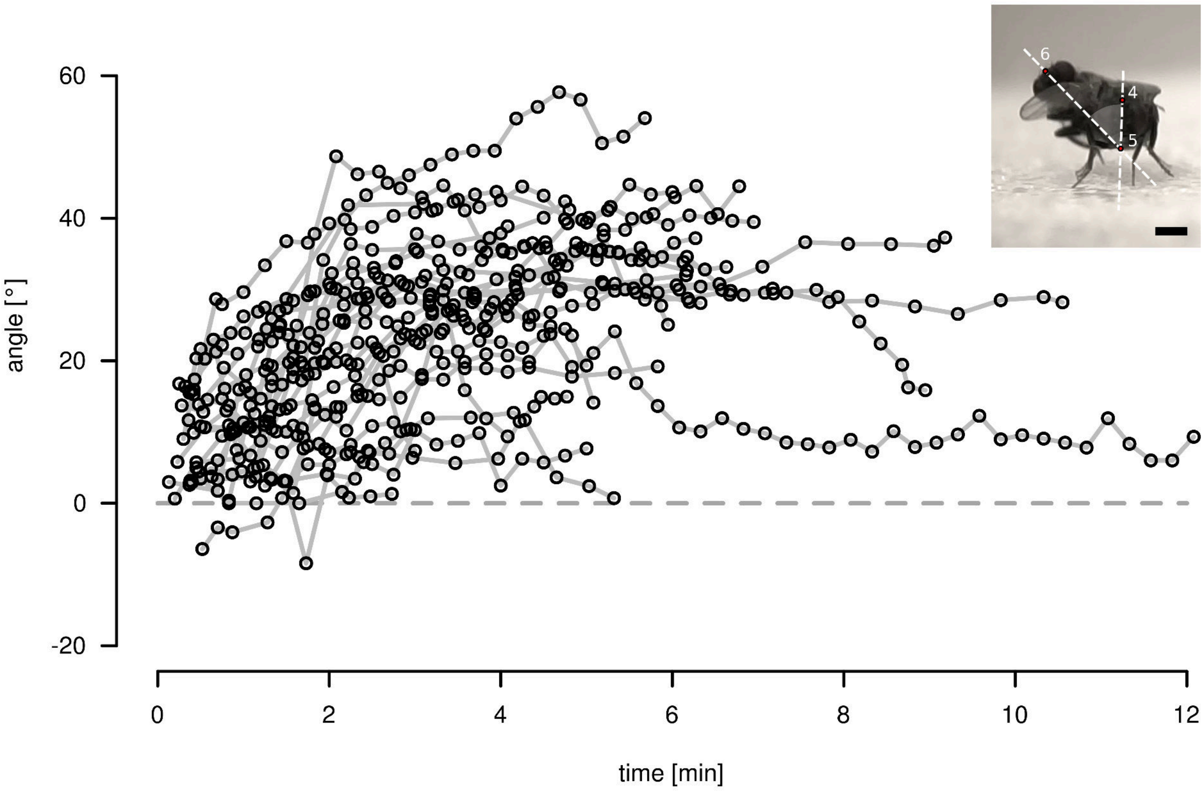

Figure S8: *D. nannoptera* tilts to the right side of the female abdomen. Frontal copulation angles (black circles) are plotted over the course of copulation. Positive and negative values indicate left-sided and right-sided angles, respectively. Grey lines connect points obtained from the same copulation couple over time. The dashed line indicates an angle of zero degrees. The position analysis from a frontal perspective is indicated on the image of the copulating couple on the right. Points and numbers indicate position landmarks P4 (4), P5 (5), and P6 (6). The dashed white lines (P1,P2) and (P2,P3) form an acute angle (semi-transparent circle sector) which corresponds to the frontal copulation angle. The scale bar is 500 µm.
