# Supplementary material for: Repeated evolution of asymmetric genitalia and right-sided mating behavior in the Drosophila nannoptera species group
Source: BMC Evol Biol. 2019 May 27;19:109. doi: 10.1186/s12862-019-1434-z (PMC6537454; doi:10.1186/s12862-019-1434-z)
Supplement: Supplementary file 3 — Multilocus DNA sequence dataset for the molecular phylogeny shown in Additional file 1: Figure S6. (PDF 59 kb) [file 12862_2019_1434_MOESM3_ESM.pdf]

```
#NEXUS
begin taxa;
	dimensions ntax=10;
	taxlabels
	D_acanthoptera[&Created=Wed Sep 12 12:16:26 CEST 2018,description=null]
	D_bromeliae[&Created=Wed Sep 12 12:16:26 CEST 2018,description=null]
	D_buzzatii[&Created=Wed Sep 12 12:16:26 CEST 2018,description=null]
	D_machalilla[&Created=Wed Sep 12 12:16:26 CEST 2018,description=null]
	D_melanogaster[&Created=Wed Sep 12 12:16:26 CEST 2018,description=null]
	D_mojavensis[&Created=Wed Sep 12 12:16:26 CEST 2018,description=null]
	D_nannoptera[&Created=Wed Sep 12 12:16:26 CEST 2018,description=null]
	D_pachea[&Created=Wed Sep 12 12:16:26 CEST 2018,description=null]
	D_willistoni[&Created=Wed Sep 12 12:16:26 CEST 2018,Modified="Tue Sep 11 22:46:08 CEST 
2018",description=null,Topology="linear","Molecule Type"="DNA"]
	D_tripunctata[&Created=Wed Sep 12 12:16:26 CEST 2018,description=null]
;
end;

begin characters;
	dimensions nchar=6813;
	format datatype=dna missing=? gap=-;
	matrix

	D_acanthoptera
GCCAATGGCGGCGATTCGGGCACTGGCGGCAGCACTGCCGATCCTGGATCCAAGGACTTCCCCGCTGTGCCATTCTCCTCGCTGGACTTTAATCCTACGT
GCGCGATTACCAACTACGCTGATCCCAGCAATGTGCGTAACTGCGAGCTGGTTGGTCTACGCGATCTGAACCAGGGCAATTCCTGGGTGCGTGACAAGAT
CGTCGATTTCCTGAACCATTTGACTGACTTGGGTGTCGCCGGATTCCGTGTGGATGCTGCCAAGCATATGTGGCCCGGAGATTTGGATGCGATTTACAGT
CGTCTGAACAATTTGAATACAGCTCATGGCTTCGATTCGGGCGCCAGACCGTACATCTTCCAGGAAGTGATCGATTTGGGTGGCGAAGCAATTTCCAAGT
CCGAGTACACTGGCATGGGTTCCGTCACTGAGTTCCGTCATTCCGACTCCATTGGCAAGGTATTCCGCGGCAAGGATCAGCTGCGTTATCTGAACAACTG
GGGCACCGCCTGGGGCTTTGCTGCCTCCGATCGCTCTCTGGTCTTTGTGGACAATCATGACAATCAGCGTGGACATGGCGCTGGTGGCGCTGATGTGCTC
ACCTACAAGGTGCCCAAGCAGTACAAGATGGCATCCGCCTTCATGTTGGCCCATCCCTTCGGCACGCCGCGTGTGATGTCCTCGTTCGCCTTCGATGACA
CCGACCAGGGACCACCGACCACCGATGGCCATAACATCGCTTCGCCCCAATTCAATGGCGACAATTCCTGCAGCGGTGGCTGGGTGTGCGAGCACCGTTG
GCGCCAGATCTACAACATGGTCGCCTTCCGCAATGCCGTGGAGAATGCCGACATCATTTACAGCCACATGCGCGACCTGAACACGGATCATGGCTTCCCC
AAGAACGCACGACCCTTCATATATCAGGAGGTCATCGATCATGGCCACGAGACTGTTTCGCGTGATGAATACACGCCTCTGGGCGCCGTCACCGAGTTCC
GTTTCTCGGAGGAAATCGGCAAGGCTTTCCGCGGCAACAATGCACTGAAGTGGCTGCAGAGCTGGGGCACCGACTGGGGCTTCATGCCCTCCGAGCAGGC
GCTCACCTTCGTGGACAACCACGACAACCAGCGCGATGGTGGTCAGGAGCTGAACTACAAGTCTCCCAAGCAGTACAAAATGGCCACCGCCTTCCATTTG
GCTTATCCCTATGGCATCAGCCAGGTGATGAGCTCGTTTGGCTTCGATGATCGCGACCAGGCGCCGCCACAGGACGCCCAAGAGCGCATCATCTCCCCGG
AATTCGATGAAGACGGCGGCTGCACCAATGGCGCCTCTGTGGAGATTGTCACTGAAACCCTCGATATCTACAATGAGTTCATGGACGCGGCCAGAGAGGA
GCACATGTGCCTCATGCACTTCAAGAGCGATGATAATGTGTACATACTCTTTGGCAACAGGATGGCCAATCACTTCAAGGAGAACGGCACGGTATTCGCC
GTGCCCATGGAACGGGCGGATCAGGTTTTTCTGGAGGAGCTGCCCAACAAGGCATTCATACTGATGGAAAACGACATCGAGCTGCGATCT---
GCTGACCTAGATCCTATGCCCACGGCCCTGGATGAGGTTCTCATTGGTAAGAGTGTGCTGCCCTCGCGGGTCCTGGTCTTCGCAAGGCCTGTCGTGGATT
TGATGAGTTCGATGAGCTATGCGAGCATGGGCTCACCCCTGGGAAACATG---
GGCGGCTGCATGGCCATGTCGGCGGCGAGCATGTCGGCGGCGGGCTTGGGCGGCAGCTATGGCTCGATGCCACCGGGCACGCGTGACATGGAGCCGGGCT
CACCGAATTCGCTTGGCCGTCGCGGCGTCGACAAGCCGACCACATACAGACGCAGCTACACGCATGCGAAGCCGCCTTACAGCTACATCTCGCTCATCAC
GATGGCCATACAGAACAATCCGACGCGCATGCTGACGCTGTCCGAGATCTATCAGTTCATCATGGATCTGTTTCCGTTCTATAGGCAGAACCAGCAGCGC
TGGCAGAACTCCATACGCCATTCGTTGAGCTTCAACGATTGCTTTGTGAAGATACCACGGACGCCGGACAAGCCCGGCAAGGGCTCGTTCTGGACGCTTC
ATCCGGACTCGGGAAATGATAATCATTGCCTCAACGCAGATGTCTTTGTGTTGGTCCTAAACGCGGAGTCCACAATGACGCGTGCGGAGAAACAATTCTT
TCATACTGTTTCACAGAAACTATCTAAACCAAATATCTTCATACTGAACAATCGCTGGGACGCCTCGGCCAATGAGCCAGAGTTCCAAGAATCGGTGAAG
TCCCAGCATACGGAACGCTGCGTGGATTTTCTCACCAAAGAGCTGAAGGTCAGCAACGAGAAGGAGGCCGCTGAGCGTGTCTTCTTCGTTTCTGCACGTG
AAACTCTGCAAGCTCGGGTCGAGGAGTCCAAGGGCAATCCGCCACATTTGGGTGCCATCGCGGATGGATTTCAGATACGCTACTTTGAGTTTCAGGACTT
TGAGCGTAAAGTACTGCCACCGATACTGCAATGCTCCAGCGGGCACTTGGTTTGCGTTTCGTGCCGCTCAAAGCTCACATGCTGCCCAACATGTCGCGGC
CCATTGGCCAATATACGTAACCTGGCCATGGAGAAGGTCGCCTCTAATGTGAAGTTCCCGTGCAAGCATTCGGGCTACGGCTGCACTGCCTCACTAGTTT
ACACAGAGAAGACCGAGCACGAGGAGACCTGCGAATGCCGGCCATACCTATGCCCCTGCCCGGGCGCCTCGTGCAAATGGCAGGGTCCGCTCGATCTAGT
CATGCAGCATCTGATGATGTCCCATAAGAGTATCACAACGCTGCAAGGCGAAGATATTGTATTTCTGGCCACCGATATAAATTTGCCCGGCGCCGTTGAT
TGGGTTATATTTAAGGAAATTGGCAGCGCCTCTAATGCTCTGCGCACCATGCAGGGCTTTCCATTCTATGACAAACCAATGCGTATTGCCTATTCCAAAT
CTGACTCGGATATTGTTGCCAAATTGAAGGGCACGTACAAAGAACGACCAAAGAAAGTGAAACCGCCAAAACCGGCACTGGGCGCTGAGGAGAAAAAGGA
CAAGAAGAAGAAGCAGAGCAGTGCCGAGAATTCCAACCCGAACACACAAACAGAACAGCCGCCAAATCAAATCCTCTTCCTCACCGCGCATGCGGTATTA
GGCAAGCACGACAATGTCGTGCGCTATTATTCTGCTTGGGCGGAGGATGATCATATGTTGATCCAAAATGAATTTTGTGATGGTGGCAGTCTGCATGCGC
GAATACAGGAGCACTGTTTAGGAGAATCCGAGCTAAAGATATTGCTAATGCATGTGATCGAAGGTTTGCGTTATATACATTCCAATGATTTGGTGCACAT
GGACATCAAACCAGAGAACATTTTCTCCACAATGAATCCGACGGCACACAAGAAAGACGATGATGGGATGGATAGTGTATATGAGGAGCTGCGCAGCTCC
GAGAACCTCGTGACGTATAAGATTGGTGACTTAGGACATGTCACATCTGTCAATGAGCCGCACGTCGAGGAAGGAGATTGTCGCTACCTGCCCAAGGAAA
TTTTGCAGGAAGACTACAGTAATCTCTTCAAGGCGGATATCTTTTCGTTGGGTATTACGCTATTCGAGGTAGCAGGCGGTGGTCCGTTGCCAAAGAATGG
ACCCGAGTGGCATAAGTTGCGCAGTGGAGAGGTACCTGCTATCCCTACGCTAAGCAAGGACTTTAACGAGTTGATTGCAAATTCTTCAAAAATTTTATTT
TTAATAATAATAATTATTGGAACATTAATTACAATTACATCAAATTCTTGGTTAGCCGCTTGAATAGGTTTAGAAATTAATTTATTATCTTTTATTCCCC
TTATAAGAGATACAAATAATTTAAAATCTACAGAAGCAGCTTTAAAATATTTTTTAACCCAAGCTTTAGCTTCAATTGTTTTATTATTTTCTATTATTTT
ATTAATATTAAAAATTAATTTAAATTATGAAATTAATTTATCGTTTATTACTACAATAATTTTGTCGTCTTTATTATTAAAAAGAGGAGCTGCTCCTTTT
CATTTTTGATTTCCTAATTTAATAGAAGGTTTAACTTGAGTAAATTCTTTTTTATTAATAACATGACAAAAAATAGCTCCATTAATACTTATATCTTATT
TAAATTTAAATAAAATACTATTTATTAGTATAATTTTATCTGTAATTGTAGGAGCAATTGGTGGTTTAAACCAAACTTCTTTACGAAAATTAATAGCTTT
TTCTTCTATTAATCATTTAGGTTGAATATTAAGTGCTTTATATATTAATGAAATAACTTGATTAATTTATTTTTTATTTTATTCATTTTTAACTTTAACA
CTTACATTTATATTTAATAATTTTAAATTATTTCATTTAAATCAATTATTTTCTTGATTCATTCAAAATAAAATTTATAAATTTATTTTATTCATTAATT
TTTTATCATTAGGAGGTTTACCTCCATTTTTAGGATTTTTACCTAAATGAATTGTTATCCAACAATTAACATTTTCAAATCAATATTTTCAATTATTTAT
TTTAATAATATCAACTTTAATTACATTATTTTTTTATTTACGAATTTGTTATTCAGCTTTTATATTGAATTATTTTGAAAATAATTGAATAAACATTCAA
TTTATAAAAAATATTTCAATAAATTTTTATTTAATTTTAACATTTTTTTCTATTTTTGGTTTATTTTTAATTCCTATAGATATTGGAACATTATATTTTA
TTTTTGGTGCATGAGCTGGAATAGTAGGAACTTCATTAAGAATTCTAATCCGAGCTGAATTAGGTCACCCAGGAGCTTTAATCGGAGATGATCAAATTTA
TAATGTAATTGTTACAGCACATGCTTTTATTATAATTTTTTTTATAGTTATACCTATTATAATTGGAGGATTTGGAAATTGATTAGTTCCTTTAATATTA
GGAGCTCCAGATATAGCTTTTCCTCGAATAAATAATATAAGATTTTGACTTTTACCTCCAGCATTAACTCTTTTATTAGTAAATAGTATAGTTGAAAATG
GAGCTGGGACAGGTTGAACTGTATACCCTCCTCTCTCAGCTGGAATCGCTCATGGAGGAGCTTCTGTAGATTTAGCTATTTTTTCTTTACATTTAGCTGG
AATTTCTTCAATTTTAGGAGCTGTAAATTTTATTACAACAGTAATTAATATACGATCAACAGGAATTACTTTAGATCGAATACCTTTATTTGTTTGATCT
GTAGTAATTACTGCTTTACTTTTATTATTATCATTACCAGTTTTAGCTGGAGCAATCACTATATTATTAACAGACCGAAATTTAAATACTTCTTTTTTTG
ATCCAGCTGGAGGAGGAGATCCTATTTTATATCAACATTTATTTTGATTTTTTGGACACCCAGAAGTTTATATTTTAATTTTACCTGGATTTGGAATAAT
```

TTCTCATATTATTAGACAAGAATCTGGTAAAAAAGAAACTTTTGGTTCTTTAGGAATAATTTATGCTATATTAGCTATTGGTTTATTAGGATTTATTGTT
TGAGCTCATCATATATTCACAGTAGGAATAGATGTAGATACTCGAGCTTATTTTACATCTGCAACTATAATTATTGCTGTTCCAACAGGAATTAAAATTT
TTAGTTGATTAGCCACTCTTCACGGTACTCAACTTTCTTACTCTCCAGCTATTTTATGAGCTTTAGGATTTGTATTTTTATTTACTGTTGGAGGTTTAAC
TGGAGTTGTATTAGCAAATTCTTCTATTGATATTATTCTTCATGATACATATTATGTCGTAGCTCATTTTCATTATGTATTATCAATAGGAGCAGTTTTT
GCTATTATAGCAGGATTTATTCATTGATATCCTTTATTTACAGGATTAACTTTAAATAATAAATGATTAAAAAGTCAATTTATTATTATATTTATTGGAG
TAAATTTAACCTTTTTTCCTCAACATTTCTTAGGATTAGCTGGAATACCACGACGATATTCTGATTATCCAGATGCTTATACATCATGAAATGTAATTTC
AACTATTGGTTCATCTATTTCTTTATTAGGAATTTTATTCTTTTTTTTTATTATTTGAGAAAGTTTAATTTCTCAACGACAAGTAATTTATCCAATTCAA
TTAAATTCTTCAATTGAATGATATCAAAATACTCCACCTGCTGAACATAGTTATTTAATAGAACAATTAATTTTTTTTCATGATCATGCATTATTAATTT
TAGTTATAATTACAGTACTTGTAGGTTATTTAATATTAACTTTATTTTTTAATAAATATATTAATCGATTTTTATTACATGGTCAATTAATTGAAGTAAT
TTGAACAATTTTACCAGCAATTATTTTATTATTTATTGCATTTCCATCTTTACGCCTATTATATCTTATTGATGAAATTAATGAACCCTCAGTAACTTTA
AAAAGAATTGGCCATCAATGATATTGAAGTTATGAATATTCTGATTTTAATAATATTGAATTTGATTCTTACATAATTCCTACAAATGAACTTAACAAAG
ATGAGTTTCGTCTCTTAGACGTTGATAATCGAATTATTCTACCAATAAATTCTCAAATTCGAATTTTAGTAACTGCAGCTGACGTAATTCATTCATGAAC
AATCCCAGCTTTAGGAGTAAAAGTTGATGGTACTCCTGGACGATTAAATCAAACCAATTTTTTTATC

D_bromeliae
GCCGATGGCGGCAACTCTGGCACTGGCGGCAGCAATGCCGATCCCAAGTCCAAGAGCTACCCGGCCGTGCCCTTCTCCTCGCTGGACTTCAACCCGACCT
GTGCCATCACCGACTACAGCAACCCCACCAATGTGCGCAACTGCGAGCTGGTTGGCCTCCGCGATCTGAACCAGGGCAACTCCTATGTGCGTGACAAGAT
TGTCGACTTCCTTAACCATCTGATCGAACTGGGTGTTGCCGGTTTCCGTGTGGATGCCGCCAAGCACATGTGGCCCGGAGATCTGGCCGTGATCTACAAC
CGCCTGAACAACCTGAACACCGATCATGGATTCGACCACGGAGCCAGGCCCTACATCGTCCAGGAGGTGATCGACTTGGGCGGCGAGGCCATCTCCAAGT
CTGAGTACACCGGCATGGGCGCCATTACCGAGTTCCGTCACTCCGACTCCATTGGCAAGGTGTTCCGCGGCAAGGATCAGCTGCGCTATCTGTCCAACTG
GGGCACCGCCTGGGGCTTCGCGGCCTCCGATCGCTCCCTGGTATTCGTCGACAACCACGACAACCAGCGTGGTCATGGCGCTGGCGGCGCTGATGTGCTC
ACCTACAAGGTGGCCAAGAAGTACAAGATGGCCAGCGCCTTCATGTTGGCCCATCCCTTCGGCGTGCCTCGCGTGATGTCCTCGTTCGCCTTCGACGACA
CCGACCAGGGCCCACCCACCACCGACGGCCACAACATTGCCTCGCCCCGCTTCAACAGCGACAACTCCTGCAGCGGCGGCTGGGTGTGCGAGCACCGTTG
GCGCCAGATCTACAACATGGTTGGCTTCCGCAACACCGTCGGCGATGCTCAGTGCATTTACAGCAACACGCGTGACCTGAACACGGATCACGGCTTCCCC
AGGAATGCTCGTCCCTTCATCTACCAGGAGGTCATTGACCATGGCCACGAGACTGTGTCGCGTGATGAATACACCCCACTCGGAGCTGTCACTGAGTTCC
GTGTTTCTGAAGAAATCGGCAAGGCTTTCCGTGGCAACAACGCACTGAAATGGCTGCAGAGCTGGGGCACCGACTGGGGCTTCCTGCCCTCCGAGCAGGC
CCTCACCTTCGTAGACAACCACGACAACCAGCGTGATGGCGGCCAGGAGTTGAACTACAAGTCTCCCCGACAGTACAAGATGGCCACTGCCTTCCATTTG
GCCTTCCCCTACGGTATCAGCCAGATCATGAGCTCCTTTGGCTTTGATGATCGCGACCAGGCACCGCCACAAGACGCCCAGGAGCGCATTATCTCGCCCG
AATTCGATGAGGATGGCGCCTGCACCAATGGCGCTTCGGTGGAGATCGTCACTGAAACCCTGGACATATACAACGAGTTTATGGACGCGGCAAGGGAGGA
GCACATGTGCCTGATGCACTTCAAGAGCGACGACAATGTCTACATACTCTTTGGTAATAAAATGGCCACCCACTTCAAGGAGAACGGAACAGTATTTGCC
GTGCCCACGGAACGGCCGGATCAGGTATTCCTAGAGGAGCTGCCAAACAAGGCATTCATTCTCATGGAGAACGACATCGAGCTGCGGACA---
GCTGACCTAGATCCAGTGCCCACAGCTTTAGACGAGCTTCTGATTGGCAAATCGGTGTTGCCCTCGCGGGTTTTGGTGTTCGCCAGTCCCGTCGTGAATC
TAATGAGCTCAATGAGCTATGCGAGCATGGGCTCACCCTTGGGCAATATG---
GGCGGCTGCATGGCCATGTCGGCGGCAAGCATGTCCGCGGCTGGCTTGAGCGGCACCTACGGCTCGATGCCACCGGGCACACGCGACATGGAGCCCGGCT
CACCGAATTCACTTGGACGTCGTGGCGTCGACAAGCCAACCACATACAGACGCAGCTACACGCACGCGAAGCCGCCTTACAGCTACATCTCCCTCATCAC
AATGGCCATACAGAACAATCCGACGCGGATGCTGACCCTCTCGGAGATCTATCAGTTCATCATGGACCTGTTCCCGTTCTACAGGCAGAACCAGCAGCGC
TGGCAGAATTCCATACGCCACTCGCTGAGCTTCAACGATTGTTTTGTGAAGATACCACGCACGCCGGACAAGCCCGGCAAGGGCTCCTTCTGGACGCTGC
ATCCGGACTCGGGCAATGATAATCATTGCCTAAATGCAGATGTCTTTGTGCTGGTCCTAAATGCTGAATCCACAATGACGCGCGCTGAGAAACAATTCTT
TCATACCGTTTCCCAGAAGCTGTCCAAGCCGAATATCTTCATACTGAACAATCGCTGGGACGCCTCGGCCAATGAGCCCGAATTTCAGGAATCGGTGAAG
TCACAGCACACGGAGCGATGTGTGGATTTCCTCACCAAGGAGCTGAAGGTCAGCAATGAAAAGGAGGCCGGAGAGCGGGTGTTCTTCGTCTCGGCACGCG
AGACGCTGCAGGCGCGCATCGAAGAGTCAAAGGGAAATCCACCGCACTTGGGTGCCATTGCCGAGGGTTTCCAGATACGCTACTTTGAGTTCCAGGACTT
TGAGCGTAAAGTGCTGCCACCGATATTGCAATGCTCCAGCGGGCACTTGGTGTGCGTCTCGTGCCGCTCAAAGCTCACATGCTGCCCAACATGCCGCGGC
CCGTTGGCCAACATACGTAATTTGGCCATGGAGAAGGTCGCCTCCAATGTGAAATTTCCGTGCAAGCATTCGGGCTACGGTTGCACTGCCTCTCTTGTTT
ACACAGAGAAGACCGAGCACGAGGAGACCTGCGAATGCCGGCCATACCTCTGTCCCTGTCCGGGCGCCTCATGCAAATGGCAGGGTCCGCTCGATCTAGT
CATGCAGCATCTGATGATGTCCCATAAGAGTATCACAACGCTGCAGGGCGAAGATATTGTATTCCTGGCCACCGATATCAATCTACCCGGCGCCGTCGAC
TGGGTCATATTTAAGGAAATTGGCAGCGCCTCTAACGCTCTGCGCACCATGCAGGGCTTCCCCTTCTATGACAAGCCAATGCGCATCGCCTACTCCAAAT
CGGATTCGGACATCGTTGCTAAACTGAAGGGCACCTACAAAGAGCGGCCGAAGAAGGTGAAACCGCCAAAGCCGGCACCGGGCACCGAGGAGAAAAAGGA
CAAAAAGAAGAAGCAGAGCAGTGCCGAAAATTCCAACCCAAACACACAGACGGAACAGCCACCAAATCAAATCCTTTTCCTCACCGCGCATGCAGTGCTT
GGAAAACACGACAACGTCGTTCGCTACTATTCGGCCTGGGCGGAGGACGACCATATGTTGATTCAAAATGAGTTCTGTGATGGCGGCAGTCTGCATGCTC
GAATTCAGGAGCATTGCCTCAGCGAATCCGATCTGAAGATATTGCTGATGCACGTAATTGAGGGTCTGCGCTATATACACTCCAATGATCTGGTGCACAT
GGACATCAAGCCGGAGAACATATTTTCCACCATGAATCCCACGGCACACAAGAAGGATGATGATGGGATGGATAGCGTGTACGAGGAGCTGCGCAGTTCC
GAGAATCTTGTCACGTACAAAATTGGTGACCTTGGGCACGTGACATCTGTTAACGAACCGTATGTCGAGGAAGGAGATTGCCGCTACCTGCCCAAGGAAA
TTCTCCAGGAGGATTATTCCAATCTTTTCAAAGCGGACATTTTCTCACTGGGAATAACGCTCTTCGAGGTGGCAGGGGGTGGACCTTTGCCAAAGAATGG
ACCTGAGTGGCACAGGCTACGAAGTGGCGAGGTGCCAACCATCCCAACGCTCAGCAAGGACTTTAACGAACTGATTGCGAACTCCTCAAAAATTTTATTT
CTTATAATAATAATTATTGGAACATTAATTACAGTTACATCAAATTCTTGGTTAGGAGCTTGAATAGGTTTAGAAATTAATTTATTATCTTTTATCCCCC
TTATAAGAGATACAAATAATTTAAAATCAACAGAAGCTTCTTTAAAATATTTTTTAACCCAAGCATTAGCTTCAACTGTTTTATTATTTTCCGTAATTTT
ATTAATATTAAAAAATAATATAAATTACGAAACAAATTCTTCATTTATTTCCATAATTACTCTTTCTACTTTATTATTAAAAAGAGGGGCCGCTCCATTT
CATTTTTGATTCCCTAATTTAATAGACGGGTTAACGTGAATAAATTCATTATTATTAATGACATGACAAAAAATTGCTCCATTAATACTAATTTCTTATT
TAAATTTTAAAAATTTATTAATAATTAGAGTAATTTTATCTGTTGTTATTGGAGCTCTTGGAGGATTAAACCAAACTTCATTACGAAAACTTATAGCTTT
TTCTTCCATTAATCATTTAGGATGAATATTAAGAAGTTTGATAATTAGTGAATCAGCATGAAGAATTTATTTTTTATTTTATAGTTTTTTATCATTTACA
TTAGCATTTATATTTAATAATTTCAAAATATTTCATTTAAATCAATTATTTTCTTGATTTATCCACAATAAAATTTTTAAATTTACTTTATTCATAAATT
TTTTATCATTAGGAGGATTACCCCCATTTTTAGGATTTTTACCTAAATGAATTGTAATTCAAGAACTTACATTTAATAATCAATATTTTTTATTAATAAT
TTTATTAATAATAACTTTAATTACTTTATTTTTTTATTTACGAATTTGTTATTCAGCATTTATACTAAATTATTTTGAAAATAACTGAATTTTAAATCAA
CAAATTAATAAAATTTCTATAAAATTTTACTTAATTTTTAGATTTTTTTCAATTTTTGGTTTATTTTTAATTTCAATAGATATTGGTACATTATATTTCA
TTTTCGGTGCATGAGCTGGAATAGTAGGTACTTCATTAAGTATTTTAATTCGAGCTGAATTAGGACATCCTGGAGCTTTAATTGGTGATGATCAAATTTA
TAATGTGATTGTTACAGCTCATGCTTTTATTATAATTTTTTTTATAGTAATACCAATTATAATTGGAGGATTTGGAAATTGATTAGTTCCATTAATATTA
GGAGCTCCTGATATAGCTTTTCCTCGGATAAATAATATAAGATTTTGATTACTACCTCCTGCTCTTTCTTTATTATTAGTAAGAAGAATAGTTGAAAACG
GAGCTGGAACAGGATGAACTGTTTATCCTCCTTTATCAGCAGGAATTGCTCATGGAGGAGCATCTGTTGATTTAGCTATTTTTTCTTTACATTTAGCTGG
AATTTCTTCAATTTTAGGGGCAGTAAATTTTATTACAACTGTTATTAATATACGATCAACAGGAATTACATTAGATCGAATACCTTTATTTGTATGATCA
GTTGTAATTACTGCTTTATTATTATTATTATCATTACCTGTTTTAGCTGGAGCTATCACAATATTATTAACAGACCGAAATTTAAATACTTCATTCTTTG
ACCCAGCGGGAGGAGGAGACCCAATTTTATATCAACATTTATTTTGATTTTTTGGACACCCTGAAGTTTATATTTTAATTTTACCTGGATTTGGAATAAT
TTCTCATATTATTAGTCAAGAATCAGGTAAAAAGGAAACATTCGGTTCTTTAGGTATAATTTATGCTATACTTGCTATTGGTTTATTAGGATTTATTGTA
TGGGCTCACCATATATTCACAGTTGGAATAGATGTTGATACTCGAGCTTATTTTACATCAGCAACAATAATTATTGCTGTTCCTACTGGAATTAAAATTT
TCAGATGATTAGCAACTCTTCATGGAACTCAACTATCTTATTCTCCTGCAATTTTATGAGCTCTTGGGTTTGTATTTTTATTCACAGTAGGGGGATTAAC
AGGTGTAGTTTTAGCTAATTCTTCTGTTGATATTATTTTACATGACACATACTATGTAGTAGCTCATTTTCATTATGTTTTATCAATAGGAGCTGTATTC
GCAATTATAGCAGGATTTATTCATTGATACCCATTATTTACTGGTTTAACTTTAAATAATAAATGATTAAAAAGTCAATTTATTATTATATTTATTGGAG
TAAATTTAACATTTTTTCCTCAACATTTTTTAGGGCTTGCTGGTATACCACGACGATACTCTGACTATCCTGATGCTTATACAACTTGAAATGTAATCTC
AACAATCGGTTCATCAATTTCATTATTAGGAATTTTATTTTTCTTTTACATTATTTGAGAAAGTTTAGTATCGCAACGACAAGTTATTTACCCAATTCAA
TTAAATTCTTCAATTGAATGATACCAAAATACACCTCCTGCTGAACATAGTTATTTAATAGAACAATTAATTTTTTTTCATGATCATGCTTTATTAATTT
TAGTAATAATTACAGTTTTACTAGGTTATTTAATACTAACTTTATTTTTTAATAATTATATTAATCGATTTTTATTACATGGACAATTAATTGAAGTAAT

TTGAACTATTTTGCCAGCTATTATTTTATTATTTATTGCTTTACCTTCTTTACGTCTTTTATATTTATTAGATGAAATTAATGAACCTTCAGTAACTTTA
AAAAGTATTGGACATCAATGATATTGAAGTTATGAATATTCAGATTTTAATAATGTTGAATTTGATTCATATATAATTCCAACAAATGAATTAGCAAATG
ACGGATTTCGTCTTTTAGATGTTGATAACCGTATTGTTTTACCTATAAATTCTCAAATTCGAATTCTTGTTACTGCCGCTGATGTAATTCATTCATGAAC
AATTCCTGCTTTAGGAGTAAAGGTAGATGGAACTCCTGGACGATTAAACCAAACTAATTTCTTTATT

D_buzzatii

GCCAATGGCGGCACCTCAGGCACTGGCGGCACCTCAGCCGATCCCAGCTCCAAGAGCTTCCCGGGCGTGCCATTCTCCTCGCTGGACTTCAATCCCACCT
GTTCAATCACCAACTACGCTGACCCCACCAATGTGCGCAACTGCGAGCTGGTTGGTCTGCGCGACTTGAACCAGGGCAACTCCTGGGTGCGTGACAAGAT
CGTTGATTTCCTGAACCATTTGACTGACCTGGGTGTTGCTGGTTTCCGTGTGGATGCTGCCAAGCACATGTGGCCCTCAGATTTGTCCGCCATCTATAAC
CGTTTGAATAACTTGAATACCGCTCATGGCTTTAGCTCGGGCGCCAGGCCTTATATCTTCCAGGAGGTCATTGATTTGGGCAACGAGGCCATCTCCAAGT
CGGAATATACCGGCCTGGGCGCTGTCACTGAGTTCCGTCATTCGGACTCCATTGGCAAGGTGTTCCGCGGCAAGGATCAGCTGCGCTATCTGCAGAACTG
GGGCACTTCCTGGGGCTTTGCCGCCTCCGATAAATCGCTTGTGTTTGTCGACAATCACGACAATCAGCGTGGACATGGCGCCGGTGGCGCCGACGTGCTC
ACCTACAAGGTGCCCAAGCAATACAAAATGGCCTCCGCCTTCATGTTGGCCCATCCGTTCGGCACACCCCGCGTGATGTCCTCCTTCGCGTTCGACAACA
CCGATCAGGGACCCCCGACCACCGATGGCCACAACATTGCCTCGCCCACATTCAACAGCGACATGTCTTGCGGCGGCGGCTGGGTGTGCGAGCATCGCTG
GCGCCAGATCTACAACATGGTTGGCTTCCGCAATGCTGTCGGTGACTCGTGGCTCATTTACAGCAACACGCGTGACCTGAACACAGACCATGGATTCCCC
AAAAACGCACGGCCCTTCATCTATCAGGAGGTCATCGACCATGGACACGAGACGGTCTCGCGTGATGAGTACACGCCGCTCGGAGCCGTCACTGAGTTCC
GTTTCTCAGAGGAAATAGGCAAGGCCTTCCGCGGCAACAATGCTCTGAAATGGCTATCGAGCTGGGGCACCGACTGGGGATTCCTGCCCTCTGACCAGGC
GCTCACCTTTGTGGACAACCACGACAATCAGCGCGACGGCGGCCAGGAGCTTAACTATAAATCGCCCAAGCAGTACAAAATGGCCACCGCCTTCCATTTG
GCCTATCCCTACGGCATCAGCCAGGTGATGAGCTCCTTCGGCTTCGACGATCGCGACCAGGCACCGCCCCAGGATGCCCAGGAGCGCATCATCTCGCCGG
AGTTCGATGAGGACGGCGGCTGTACCAATGGCGCCTCAGTGGAGATTGTCACAGACACTTTGGATGTATACAACGAGTTCATGGATGCCGCCAGGCAGGA
GCACATGTGCTTGATGCACTTCAAGAGCGACGACAATGTTTACATACTTTTCGGCAACAAGATGGCCAATCACTTCAAGGAAAACGGCACGGTATTTGCC
GTGCCCACAGAGCGTGCAGATCATGTATTCCTGGAGGAGCTGCCCAATAGGGCATTCGTACTGATGGAGAATGAGATAGAGCTGCGAGCG---
GCTGACCTGGATCCCATGCCCACGGCACTCGACGAGGTGCTCGTAGGCAAGAGTGTGCTGCCCTCGCGCGTCCTTGTCTTTGCCAGCCCCGTTGTTGATC
TGATGAGTTCGATGAGCTACGCTAGCATGGGCTCGCCACTGGGCAACATG---
GGCGGCTGCATGGCCATGTCGGCGGCGAGCATGTCGGCGGCTGGCTTAGGCGGCAGCTATGGCTCGATGCCACCAGGCACCCGAGACATGGAGCCCGGCT
CACCCAATTCACTTGGAAGACGAGGCGTTGACAAGCCAACTACATACAGACGCAGCTACACGCACGCGAAGCCGCCTTACAGCTACATCTCGCTCATCAC
GATGGCCATTCAGAACAATCCCACACGCATGCTAACATTGTCGGAGATCTATCAGTTCATCATGGATCTGTTTCCGTTCTATCGGCAGAATCAGCAGCGC
TGGCAGAACTCCATACGCCACTCACTGAGCTTCAACGACTGCTTTGTGAAGATACCACGAACGCCGGATAAGCCCGGCAAAGGCTCCTTCTGGACGCTGC
ATCCGGACTCGGGAAATGACAATCATTGCATTAATGCGGATGTTTTTGTGCTGGTGCTCAATGCGGAGTCGACAATGACACGCGCTGAGAAGCAGTTCTT
TCACACTGTTTCCCAGAAGCTGTCCAAGCCAAACATCTTCATACTGAACAATCGCTGGGATGCGTCGGCCAATGAGCCCGAGTTTCAAGAATCGGTCAAG
TCACAGCACACGGAGCGTTGTATCGATTTCCTCACCAAGGAGCTGAAGGTCAGCAACGAGAAGGAGGCGGCGGAGCGTGTGTTCTTTGTGTCGGCGCGCG
AGACGCTGCAGGCGCGCATGGAGGAGTCAAAGGGCAATCCGCCGCATCTGGGCGCCATTGCTGAGGGCTTTCAGATACGCTACTTTGAGTTCCAGGACTT
TGAGCGCAATGTGCTGCCACCGATATTGCAATGCTCGAGCGGACATTTGGTCTGCGTCTCGTGCCGATCAAAGCTCACATGCTGCCCCACATGCCGCGGC
CCCTTGGCCAACATACGCAATCTGGCCATGGAAAAGGTCGCCACCAATGTTAAGTTTCCATGCAAGCATTCGGGCTACGGCTGCACTGCCTCACTCGTTT
ACACAGAGAAGACCGAGCACGAGGAGACCTGCGAATGCCGGCCCTATCTATGTCCCTGTCCGGGTGCATCATGCAAATGGCAGGGACCGCTCGATCTAGT
CATGCAGCATCTGATGATGTCCCACAAGAGCATTACCACGCTGCAGGGCGAGGATATTGTCTTTCTGGCCACCGACATCAATCTGCCCGGCGCCGTCGAC
TGGGTGATATTTAAAGAAATTGGCAGTGCCTCCAATGCGCTGCGTACAATGCAGGGCTTTCCGTTCTATGACAAGCCCATGCGCATTGCCTACTCCAAGT
CCGATTCGGATATTGTGGCCAAATTGAAGGGCACCTACAAGGAGCGGCCAAAGAAGGTGAAGCCGCCAAAGCCGGTGCCGGGTGTCGAGGAGAAAAAGGA
CAAGAAGAAGAAGCAGAGCAGTGCGGAAAATTCAAATCCGAATACACAAACAGAACAGCCACCAAATCAAATCCTTTTCCTCACCGCTCATGCGGTGTTG
GGCAAGCACGACAATGTGGTGCGCTACTATTCGGCCTGGGCGGAGGACGATCATATGTTGATACAGAATGAATTTTGTGATGGCGGTAGTCTGCATGCGC
GCATCCAAGAGCACTGCCTGGGTGAATCTGAGCTGAAGATCTTACTGATGCATGTGATCGAAGGCCTGCGATATATACATTCCAATGATTTGGTGCACAT
GGACATCAAGCCGGAGAATATCTTCTCAACCATGAATCCCACCGCGCACAAGAAAGATGATGATGGCATGGATAGCGTTTACGAGGAGCTACGCAGCTCT
GAGAATCTCGTAACATACAAAATTGGTGACTTAGGACATGTGACGTCCGTGAACGAGCCATACGTCGAGGAGGGCGATTGCCGCTACCTGCCCAAAGAGA
TACTGCAGGAAGATTATAGCAATCTGTTCAAGGCGGACATCTTCTCGCTAGGTATCACCCTCTTCGAGGTAGCTGGTGGCGGACCGCTGCCAAAGAACGG
GCCCGAGTGGCACAAGTTGCGCAATGGCGAAGTGCCAATCATACCAGCGTTGAGCAAGGACTTTAATGAGCTGATCGCTAACTCTTCAAAAATTTTATTT
TTTACAATAATAATTATTGGAACTCTAATCACAGTAACCGCAAATTCTTGGTTAGGAGCTTGAATAGGTTTAGAAATTAATTTATTATCTTTTATCCCCC
TTATAAGAGATAGTAATAATTTAAAATCAACTGAAGCTTCTTTAAAATATTTTTTAACCCAAGCTCTTGCTTCAACAGTTCTTTTATTTTCCGTAATTTT
ATTAATATTAGAAAATAGCATAAATTATGAAATTAATAGTTCTTTTATTTCAATAATTACATTATCAACTTTATTATTAAAAAGAGGAACAGCTCCATTT
CATTTTTGATTTCCAAATTTAATAGACGGATTAACTTGAATAAATGCTTTACTTTTAATAACGTGACAAAAAATTGCACCTTTAATATTAATTTCTTATC
TAAATATAAAAGATATTTTATTAATTAGAGTAATTTTGTCAGTTATAATTGGAGCAATTGGAGGTTTAAACCAAACATCGCTACGTAAATTAATAGCTTA
TTCTTCAATTAATCATTTAGGTTGGATATTGAGAGCTTTAATATTAAATGAATCCGCTTGATTTATTTATTTTTTATTTTATTCTTTTTTATCATCTATT
TTAATTTTTATATTTAATATATTCAAATTATTTCATTTAAATCAGTTATTTGTTATATTTACACAAAGAAAAATTTTAAAATTTATTTTATTTATAAATT
TTTTATCCTTAGGGGGTTTACCACCATTTTTGGGATTTTTACCAAAATGAATTGTAATCCAACAACTAACTTTTTGTAGCCAATACTTTCAATTATTAAT
TTTATTACTATCAACTTTAATCACATTATTTTTTTATCTACGAATATGTTATTCTGCTTTTATGCTAAATTATTACGAAAACAATTGAAATATAAGCTTA
CAAATTAATAACTTTTTAATAAACATTTATATAATCTTTTCATTTATTTCAATTTTTGGGTTAATATTTATCCCAATAGATATTGGAACTTTATATTTTA
TTTTCGGAGCCTGAGCCGGAATAGTAGGAACATCTTTAAGAATTTTAATTCGTGCTGAATTAGGTCATCCTGGAGCTTTAATTGGTAACGACCAAATTTA
TAATGTAATTGTAACAGCTCACGCTTTTGTAATAATTTTTTTTATAGTTATACCTATTATAATTGGAGGATTTGGAAATTGATTAGTACCATTAATATTA
GGAGCTCCTGATATAGCATTCCCTCGAATAAACAATATAAGTTTTTGACTATTACCTCCTGCTTTAACACTTTTGTTAGTAAGTAGTATAGTGGAAAATG
GAGCTGGAACAGGATGAACTGTTTACCCTCCCCTATCTTCTGGTATTGCACATGGTGGTGCTTCAGTTGATTTAGCTATTTTCTCTTTACACTTAGCCGG
AATTTCTTCAATTTTAGGGGCAGTAAATTTTATCACAACAGTAATTAATATACGATCTACAGGAATTACATTAGACCGAATACCTTTATTTGTTTGATCA
GTAGTAATTACTGCTTTATTGCTTCTCCTATCTTTACCAGTTTTAGCCGGAGCAATTACTATACTTTTAACTGACCGAAATTTAAATACTTCTTTTTTCG
ACCCGGCAGGAGGAGGAGATCCAATTCTTTACCAACATTTATTTTGATTTTTTGGACACCCAGAAGTTTATATTTTAATTTTACCTGGATTTGGAATGAT
TTCTCATATTATCAGCCAAGAATCAGGAAAAAAAGAAACATTTGGGTCTTTAGGAATAATTTACGCAATACTAGCAATTGGATTACTTGGATTCATTGTT
TGAGCCCATCATATATTTACAGTTGGTATAGACGTAGATACACGAGCTTATTTTACATCAGCAACAATGATTATTGCAGTCCCAACAGGAATTAAAATTT
TTAGATGACTTGCCACTTTACACGGAACTCAACTTTCTTACTCACCAGCTATTCTATGAGCTTTAGGATTTGTATTTTTATTCACAGTTGGAGGTTTAAC
TGGAGTAGTTCTCGCAAACTCATCAATTGATATTATTTTACATGATACATATTATGTAGTAGCTCACTTTCATTATGTTCTTTCAATAGGAGCAGTATTT
GCTATTATAGCAGGATTCATTCATTGATACCCTTTATTCACAGGATTAACTTTAAATAATAAATGATTAAAAAGTCAATTTATCATTATATTCATTGGTG
TAAATTTAACTTTTTTCCCTCAACACTTTCTAGGATTAGCAGGTATGCCTCGACGATACTCAGATTACCCAGATGCTTACACAACATGAAATGTAATTTC
AACAATCGGTTCATCAATCTCATTATTAGGAATTTTATTTTTCTTTTATATTATTTGAGAAAGTTTAGTATCACAACGACAAGTGATTTACCCTATTCAA
TTAAATTCTTCAATCGAATGATATCAAAATACTCCTCCCGCTGAACACAGATACTTAATAGAACAACTAATTTTTTTTCACGATCATGCTTTATTAATTT
TAGTAATAATTACTGTTTTAGTAGGATACCTTATATTCATATTATTTTTTAATAATTATGTAAATCGATTTCTTCTCCACGGGCAATTAATTGAAATAAT
TTGAACTATTTTACCAGCAATTATTCTTTTATTTATTGCATTCCCTTCTTTACGATTACTTTACCTTCTTGATGAAATTAATGAACCTTCGGTTACTCTA
AAAAGAATTGGTCACCAATGATATTGATCTTATGAATATTCAGATTTTAATAATATTGAATTTGATTCTTATATAATCCCTACAAATGAACTAACAACTG
ATGGATTTCGTTTACTAGATGTTGATAATCGAATTGTACTTCCAATAAATTCTCAAATTCGAATTTTAGTAACAGCCGCAGATGTAATTCATTCTTGAAC
AATCCCAGCTTTAGGAGTAAAAGTTGATGGAACACCTGGGCGTTTAAACCAAACTAATTTTTTCATC

D_machalilla

GCCAATGGCGGCACTTCGGGCACTGGCGGCTCCTCAGCCAATCCCAGCTCGAAGAGCTTCCCGGCTGTGCCATTCTCCTCGCTCGACTTCAATCCGACCT
GCGCCATCACTAACTATGCKGATCCCAACAATGTGCGCAACTGCGAGCTGGTTGGCCTGCGCGATCTGAACCAGGGCAACGCTTATGTGCGTGACAAGAT
TGTCGACTTCCTGAACCATTTGATCAACTTGGGTGTTGCCGGTTTCCGTGTGGATGCCGCCAAGCACATGTGGCCCGGAGACTTGTCTGCCATCTACAAC

CGGCTGAACAACCTGAACACAGATCATGGCTTCAACTCTGGTGCCAGGGCCTACATCTTCCAGGAGGTCATCGATTTGGGCGGCGAAGCCATCTCCAAGT
CCGAGTACACCGGCCTGGGCGCCGTCACTGARTTCCGTCACTCCGATTCCATTGGCAAGGCTTTCCGCGGTAAGGATCAACTGCGCTACCTGAATAACTG
GGGCACTGCCTGGGGCTTCGCTGCCTCTGACCGCTCCCTGGTCTTTGTGGACAACCACGACAACCAGCGTGGACATGGCGCCGGTGGCGCTGATGTGCTC
ACCTACAAGGTGCCCAAGCAGTACAAGATGGCCTCCGCTTTCATGTTGGCCCATCCCTTCGGYACACCCCGCGTGATGTCCTCGTTCGCCTTCGATAACA
CCGACCAGGGACCACCGACCACCGATGGCCACAACATCGCCTCRCCCAGCTTCAAGAGCGATAACTCCTGCAACGGCGGCTGGGTGTGCGAACACCGCTG
GCGCCAAATCTACAACATGGTTGCCTTCCGCAACGCCGCCAACAATGCCCAGCTGATTTACAGCAATATGCGTGACCTGAACACAGATCATGGCTTCCCC
AAGAAYGCTCGTCCGTTCATCTACCAGGAGGTSATCGATCACGGACACGAAACCGTGTCGCGTGATGAATACACTCCACTGGGCACCGTCACCGAGTTCC
GTTTCTCGGAAGAAATCGGCAAGGCCTTCCGCGGCAACAATGCACTYAAATGGCTGCAGAGCTGGGGCACTGACTGGGGCTTCCTGCCCTCCGAGCTGGC
GCTCACATTTGTGGACAACCACGACAACCAGCGTGATGGCGGCCAGGAGTTGAACTACAARTCYCCCAAGCAGTACAAAATGGCCACCGCCTTCCACTTG
GCCTATCCCTATGGCATCAGCCAGGTGATGAGCTCGTTTGGCTTTGACGATCGCGACCAGGCGCCGCCCCAGGATGCCCARGAGCGTATCATCTCGCCCG
AATTCGATGAGGATGGCGGCTGCAGCAACGGCGCCTCTGTGGAAATAGTCACTGAAACMCTGGAYACATACAACGAGTTCATGGATGCGGCCCGAGAGGA
GCACATGTGCCTGATGCACTTCAAGAGCGACGATAACGTCTACATACTCTTCGGCAACAAGATGGCCAATCACTTCAAGGAGAACGGCACAGTATTCGCT
GTGCCCACAGAGCGGGCGGACCAGRTCTTTCTGGAGGAGCTGCCCAACAAGGCTTTCATACTTATGGAGAACGAAATCGAGCTGCGTTCA---
GGCGACCTGGACCCCATGCCYACTGCTCTGGACGAGGTTCTCATTGGCAAAAATGTGTTACCCTCACGCATCTTGGTCTTCGCCAATCCCGTTGTGGATC
TAATGAGTTCTATGAGCTACGCCAGCATGGGCTCACCATTGGGCAACATG---
GGTGGCTGTATGGCCATGTCCGCGGCGAGCATGTCGGCGGCTGGCCTGGGTGGCGGYTATGCCTCGATGCCACCTGGCACTCGCGACATGGAGCCCGGCT
CTCCGAATTCCCTGGGACGTCGCGGTGTCGATAAGCCGACCACATACAGACGCAGCTATACGCACGCGAAGCCGCCTTACAGCTACATCTCGCTCATCAC
CATGGCCATACAGAACAATCCGACGCGSATGCTGACGCTTTCGGAGATATATCAGTTCATCATGGATCTGTTCCCGTTCTACAGGCAGAACCAGCAACGT
TGGCAAAACTCCATACGCCACTCACTGAGCTTCAACGATTGTTTTGTGAAGATACCACGCACACCGGACAAGCCCGGCAAAGGGTCGTTCTGGACGCTTC
ATCCGGATTCGGGAAATTGACACCATTGTCTCAACGCAGATGTCTTTGTATTGGTCCTGAATGMMGAGTCGACGATGACGCGCGCCGAGAAACAATTTTT
TCACACCGTTTCCCAGAAACTCTCTAAGCCGAACATCTTTATACTCAAYAATCGATGGGACGCTTCGGCCAATGAGCCGGAATTTCAGGAATCGGTGAAG
TCGCAGCATACGGAACGCTGCGTGGATTTCCTCACCAAGGAGCTGAAGGTCAGCAACGAAAAGGAGGCCGCCGAGCGTGTGTTCTTTGTGTCCGCACGCG
AGACGCTGCAGGCACGCATYGAAGAGTCCAAGGGTAATCCGCCGCATTTGGGTGCCATTGCCGAAGGATTTCAGATACGCTACTTTGAGTTCCAGGACTT
CGAGCGTAAAGTGCTGCCACCGATATTGCAATGCTCCAGCGGTCACTTGGTGTGCGTCTCGTGCCGCTCAAAGCTCACATGCTGCCCAACATGCCGCGGC
CCATTGGCCAACATACGTAACCTGGCCATGGAGAAGGTCGCCTCCAATGTGAAGTTTCCATGCAAGCATTCGGGCTACGGCTGTACTGCCTCGCTTGTTT
ACACAGAGAAGACCGAGCACGAGGAGACCTGCGAATGCCGGCCATACCTTTGTCCCTGCCCGGGTGCCTCATGCAAATGGCAGGGACCGCTCGATCTAGT
CATGCAGCATCTGATGATGTCCCATAAGAGTATCACAACGCTGCAAGGCGAAGATATTGTATTTCTGGCCACCGATATAAATCTGCCCGGCGCCGTCGAC
TGGGTCATATTTAAGGAAATTGGCAGTGCCTCCAATGCTCTGCGCACTATGCAGGGCTTTCCATTTTATGACAAACCAATGCGTATTGCCTATTCAAAAT
CGGACTCCGATATTGTTGCAAAATTGAAGGGCACCTACAAAGAGCGTCCAAAGAAGGTCAAACCGCCAAAACCGGCACCCGGCGCCGAGGAGAAAAAGGA
CAAGAAGAAGAAGCAGAGCAGTACCGAGAATTCCAACCCGAACACGCAAACGGAACAACCACCAAATCAAATCCTTTTCCTCACCGCGCATGCGGTGCTG
GGTAAGCACGACAATGTCGTTCGGTACTATTCGGCTTGGGCGGAGGACGATCATATGCTAATACAAAATGAATTTTGCGATGGAGGCAGTCTGCATGCAC
GTATACAGGATCATTGCTTGGGTGAATCCGAGCTAAAGATATTGCTAATGCATGTGATCGARGGTCTACGSTATATACATTCCAACGATCTGGTACACAT
GGACATCAAGCCAGAGAACATTTTCTCCACCATGAACCCTATGGCACACAAGAAAGACGATGATGGGATGGATAGTGTATACGARGAGCTGCGTAGTTCC
GAGAATCTCGTCACGTATAAGATTGGTGACTTGGGACACGTGACATCTGTGAAGGAGCCGCACGTCGAGGAAGGRGATTGCCGCTACCTGCCTAAGGAAA
TTCTGCAGGAGGATTACAGCAATCTMTTTAAGGCGGATATCTTTTCGCTAGGTATAACCCTGTACGAGGTAGCRGGCGGTGGACCGCTACCAAAAAATGG
CCCTGAGTGGCATAAGTTGCGCAGTGGCGATGTGCCAGTTATACCCACGTTAAGCAAGGACTTCAATGAACTGATTGCGAATTCTTCAAAAATTTTGTTT
TTTATAATAATAATCACAGGAACATTAATTACAGTTTCGTCTAATTCATGGTTAGGAGCTTGAATAGGTTTAGAAATTAATTTATTATCTTTTATTCCCC
TTATAAGAGATACAAATAATTTAAAATCTACAGAAGCTTCTCTAAAATATTTTTTAACCCAAGCATTAGCCTCAACTGTTTTATTATTTTCTGTTATTTT
ACTTATATTAAAAAATAATTTAAATTATGAATTTAATTTTTCATTTATCACATTAATAATCTTATCTTCTTTATTATTAAAAAGAGGAGCGGCACCCTTT
CATTTTTGATTCCCAAACCTAATAGAAGGTTTAACATGAATTAATGCTTTATTAATTATGACCTGACAAAAAATTGCTCCTTTAATATTAATTTCTTATT
TAAATTGTAGAAATTTTTTATTAATTAGAGTAATTCTTTCTGTTACTATCGGGGCATTGGGGGGATTAAACCAAACTTCTCTTCGAAAATTAATAGCTTT
TTCTTCAATTAATCATCTTGGATGACTACTAAGAGCAATTTCTATTAATGAAAATGTATGAATTTTCTATTTTATATTTTATTCTTTTTTAACATTTACT
TTAACTTTTAAATTTAATAATTTTAAATTATTTCATTTAAATCAATTATTTTCTTGATTTACACAAAATAAAATTTTTAAATTTACATTATTTATAAATT
TTTTATCATTAGGGGGTTTGCCTCCCTTTTTAGGATTTTTACCAAAATGAATCGTAATCCAACAATTAACATTTATAAATCAATATTTTCAATTATTCAT
TTTGTTATTATCAACTTTAATTACCTTATTTTTTTACCTACGAATTTGTTATTCAGCATTTATACTTAATTACTATGAAAATAATTGAAATTCAATTCAA
TATACAAATAATTTTTTATTAAAATTATACTTATTGCTTTCATTTTTCTCAATTTTTGGATTATTTTTAGTTTCTATAGATATTGGAACTTTATATTTTA
TTTTTGGAGCTTGAGCAGGAATAGTAGGAACTTCATTAAGAATTTTAATTCGTGCTGAATTAGGGCACCCAGGAGCTTTAATTGGTGATGACCAAATTTA
TAATGTAATTGTTACAGCTCATGCTTTTGTTATAATTTTTTTTATAGTAATACCTATTATAATTGGGGGATTCGGTAATTGATTGGTACCTTTAATACTA
GGAGCCCCAGATATGGCATTCCCACGAATAAATAATATAAGATTTTGACTTTTACCTCCTGCTTTAACATTATTGTTAGTAAGCAGTATAGTTGAAAATG
GAGCAGGTACAGGGTGAACGGTTTACCCTCCTTTATCAGCAGGGATTGCTCATGGAGGAGCTTCTGTTGATTTAGCTATTTTTTCTCTCCATTTAGCTGG
TATCTCTTCAATTCTAGGAGCTGTTAATTTTATCACAACTGTAATTAATATACGTTCAACAGGGATTACCCTTGACCGTATGCCTTTATTCGTTTGATCT
GTAGTAATTACAGCTTTACTATTACTTTTATCATTACCTGTATTAGCTGGAGCAATCACAATACTTTTAACAGACCGAAATTTAAACACCTCTTTTTTTG
ATCCTGCCGGAGGAGGAGACCCAATTCTTTACCAACATTTATTTTGATTCTTCGGGCACCCAGAAGTTTATATTCTTATTTTACCTGGATTTGGAATAAT
TTCTCATATTATTAGTCAAGAATCAGGGAAAAAGGAAACATTTGGATCTTTAGGGATAATCTATGCTATACTAGCAATTGGTTTATTAGGATTTATTGTC
TGAGCTCATCATATATTTACAGTTGGAATAGATGTAGACACTCGGGCATATTTTACATCTGCCACAATAATTATTGCTGTTCCAACAGGAATTAAAATTT
TTAGATGATTAGCAACTTTACATGGAACTCAACTATCTTACTCCCCAGCTATCTTATGAGCATTAGGATTTGTTTTCCTTTTTACTGTTGGAGGTTTAAC
AGGGGTTGTTTTAGCAAATTCTTCAGTTGATATTATTCTTCATGATACATATTATGTAGTAGCTCATTTTCATTATGTTTTATCAATAGGAGCTGTATTT
GCTATTATAGCGGGATTTATCCACTGATACCCTTTATTTACTGGATTAACATTAAATAATAAATGATTAAAAAGTCAATTTATCATTATATTTATTGGAG
TAAATTTAACATTTTTCCCTCAACATTTTTTAGGATTAGCAGGTATACCCCGCCGTTATTCTGATTATCCTGATGCTTATACAACTTGAAATGTAATTTC
AACAATCGGTTCATCAATTTCTTTACTTGGAATTTTATTCTTTTTTTTTATCATTTGAGAAAGATTAGTATCCCAACGTCAAGTAATTTACCCAATTCAA
TTAAATTCATCAATTGAATGATACCAAAATACCCCTCCTGCTGAACATAGCTATTTAATAGAACAATTAATTTTTTTTCACGATCATGCTTTATTAATTT
TAGTAATAATTACAGTATTAGTAGGTTATTTAATATTAACTTTATTTTTTAATTCTTATATTAATCGATTCTTATTGCATGGTCAATTAATTGAAGTAAT
TTGAACAATTTTACCAGCTATTATTTTATTATTTATTGCTTTCCCATCTTTACGTTTATTATATCTTTTAGATGAAATTAATGAACCTTCAGTTACTCTA
AAAAGTATTGGTCATCAATGATACTGAAGTTATGAATACTCTGATTTTTCAAATATTGAATTCGATTCATATATAATTCCAACAAATGAATTAAATACAG
AAGGTTTTCGTTTATTAGATGTTGATAACCGAATTATTCTTCCGATAAACTCACAAATTCGAATTTAGTAACCGCTGCCGATGTAATTCATTCATGAAC
AATCCCAGCTTTAGGTGTAAAAGTTGATGGGACCCCTGGTCGACTAAATCAAACTAGTTTTTTTATC

D_melanogaster
GCCGACGGAGGCACCTACGGCACTGGCGGCAGCACCGCCAGCCCCAGCAGCAAGAGCTATCCCGGAGTGCCCTACTCCTCGCTGGACTTCAACCCGACCT
GCGCCATCAGCAACTACAACGACGCCAACGAGGTGCGCAACTGCGAGCTGGTCGGTCTGCGCGACCTTAACCAGGGCAACTCCTACGTGCAGGACAAGGT
GGTCGAGTTCCTGGACCATCTGATTGATCTCGGCGTGGCCGGATTCCGCGTGGACGCCGCCAAGCACATGTGGCCCGCCGACCTGGCCGTCATCTATGGC
CGCCTCAAGAACCTGAACACCGACCACGGCTTCGCCTCGGGATCCAAGGCGTACATCGTCCAGGAGGTCATCGACATGGGCGGCGAGGCCATCAGCAAGT
CCGAGTACACCGGACTGGGCGCCATCACCGAGTTCCGCCACTCCGACTCCATCGGCAAGGTCTTCCGCGGCAAGGACCAGCTGCAGTATCTGACCAACTG
GGGCACCGCCTGGGGCTTCGCCGCCTCCGACCGCTCCCTGGTATTCGTCGACAACCACGACAACCAGCGCGGACATGGAGCAGGAGGCGCCGACGTGCTG
ACCTACAAGGTGCCCAAGCAGTACAAGATGGCCTCCGCCTTCATGCTGGCGCACCCATTCGGCACTCCCCGCGTGATGTCCTCCTTCTCCTTCACGGACA
CCGATCAGGGCCCGCCCACCACCGACGGCCACAACATCGCCTCGCCCATCTTCAATAGCGACAACTCCTGCAGCGGCGGCTGGGTGTGCGAGCACCGCTG
GCGCCAGATCTACAACATGGTGGCCTTCCGGAACGCCGTGGGCTTGGACGAGATCATCTACAGCAGCCTGAGCAACCTGAACATCGATCACGGCTTTCCT
CACAACTCCCGTCCCTTCATCTTCCAGGAGGTAATTGATCACGGTCACGAAACCGTCTCCCGTGATGAGTACAAGGATCTGGGTGCCGTCACCGAGTTCC
GATTCTCCGAGGAGATTGGCAACGCCTTCCGTGGCAACAACGCACTGAAATGGCTGCAGAGCTGGGGAACCGATTGGGGATTCTTGCCCTCGGGTCAGGC
CTTGACCTTTGTGGACAACCACGATAACCAGCGAGACGCGGGTGCCGTGCTGAACTACAAGTCCCCCAGGCAGTACAAAATGGCAACCGCCTTCCATTTG

GCCTATCCCTACGGCATCAGTCGGGTGATGAGCTCATTCGCCTTCGATGACCACGATACCCCGCCGCCGCAGGATGCGCAGGAGAGAATAATTTCACCCG
AGTTCGATGCGGATGGAGCCTGTGTGAATGGCGCATCCGTGGAAATAGCAACCGAAACGCTGGAGATGTACAATGAATTCATGGAAGCCGCTCGCCAGGA
GCACATGTGCCTCATGCACTTCAAAAGTGACGATAATGTTTACATAATGTTTGGCAATAAGCTAGCTAGTCACTTCAAGGAAAACGGCACTCTGTTTTCC
GTGCCCACCGATAGAACGGACGATGAATTTCTGGCAGACTTACCAAACAGAGCTTTTGTCCTAATGGAAAACGAAATTGACCTGAGCACCGCCGTTGAAT
TGGACGCCACGCCCACCGCTTTGGACGAGATCCTGATCGGGAAGAGTGTGTTACCATCGCGAGTCCTGAGCTTTGCCGGCTCCATAATCGACCTGATGAG
CTCGATGAGCTACGCCAGCATGGGATCACCGCTCGGGAACATG---
GGTGGCTGCATGGCCATGTCGGCAGCGAGTATGTCGGCGGCGGGATTGAGCGGCACCTATGGCGCCATGCCGCCGGGATCCCGGGAAATGGAGACGGGAT
CGCCGAATTCCCTAGGCAGATCGCGGGTGGACAAACCAACTACGTACAGAAGGAGCTACACGCATGCCAAGCCGCCATACAGCTACATCTCACTGATCAC
CATGGCCATTCAGAATAACCCCACCAGAATGTTGACGCTCTCGGAGATCTATCAGTTCATCATGGATCTGTTTCCGTTCTACAGGCAGAATCAGCAGCGC
TGGCAGAACTCCATTCGACATTCGCTGAGCTTCAACGATTGCTTCGTAAAGATTCCCAGGACGCCGGACAAGCCAGGAAAGGGATCGTTTTGGACTCTGC
ATCCGGATTCGGGGAATGATAACCATTGCCTGAACGCCGATGTCTTTGTGCTGGTCCTGAATGCCGAGTCAACGATGACGCGTGCGGAGAAGCAGTTCTT
TCACACCGTCTCGCAGAAGCTAAGCAAGCCGAACATCTTCATCCTGAACAATCGCTGGGATGCGTCGGCCAACGAGCCCGAGTGCCAGGAATCGGTTAAG
TCTCAGCACACGGAACGCTGCATCGACTTCCTCACCAAGGAGCTAAAGGTGAGCAACGAGAAGGAGGCGGCCGAAAGGGTATTCTTCGTTTCCGCCAGGG
AAACGCTGCAGGCGCGCATCGAGGAGGCCAAGGGCAATCCGCCGCACATGGGTGCCATTGCCGAGGGCTTTCAGATACGCTACTTCGAGTTCCAGGACTT
CGAGCGCAAGGTGCTACCGCCGATCCTGCAGTGCTCCAGCGGGCACCTGGTGTGCGTGTCCTGTCGCTCCAAGCTCACCTGCTGCCCCACCTGCCGCGGT
CCGCTGGCGAATATCCGCAACCTGGCGATGGAGAAGGTTGCCTCGAACGTCAAGTTCCCGTGCAAGCACTCCGGCTACGGATGCACCGCCTCGCTGGTTT
ACACGGAAAAGACAGAACACGAGGAGACGTGCGAGTGCCGGCCCTACCTGTGTCCGTGCCCGGGCGCCAGCTGTAAGTGGCAGGGCCCGCTTGACCTAGT
CATGCAGCATCTGATGATGTCGCACAAGAGCATCACAACGCTGCAGGGCGAGGACATCGTGTTCCTGGCCACCGACATCAACCTGCCCGGCGCCGTCGAT
TGGGTAATCTTCAAGGAGATCGGCAGCGCTTCGAATGCCCTGCGCACCATGCAGGGCTTCCCGTTCTACGACAAGCCCATGCAGATCGCCTACTCCAAAT
CCGATTCGGATATTGTGGCCAAGATAAAGGGTACCTTCAAGGAGCGCCCCAAGAAGGTCAAGCCACCAAAACCAGCGCCGGGTACCGATGAGAAGAAGGA
CAAGAAGAAGAAGCCGAGCAGCGCCGAGAACTCGAACCCGAACGCACAGACCGAGCAGCCGCCGAACCAGATCCTCTTCCTCACCGCCCATGCTGTATTG
GGCAAGCACGATAACGTGGTTAGATACTATTCCGCGTGGGCGGAGGACGATCATATGCTGATACAGAATGAATTCTGCGATGGTGGCAGCTTGCACGCGC
GCATCCAAGATCATTGTTTGGGTGAGGCAGAGCTAAAGATTGTTCTCATGCATGTGATAGAGGGCCTACGCTATATTCATTCAAATGACCTGGTGCACAT
GGATCTGAAGCCCGAAAACATATTCTCCACCATGAATCCCAATGCACACAAGAAAGATGACGATGGCATGGACAGCGTCTATGAAGAACTGCGCCATTCA
GAGAATCTGGTTACGTACAAGATCGGCGACCTGGGACACGTGACCTCCGTTAAGGAACCCTACGTCGAGGAGGGAGACTGTCGTTACCTGCCCAAGGAGA
TCCTTCACGAGGATTATTCGAATCTCTTCAAGGCCGACATATTTTCCCTGGGCATCACGCTGTTCGAGGCGGCCGGCGGTGGTCCGCTGCCTAAAAATGG
ACCCGAATGGCACAACTTGCGAGATGGCAAGGTGCCGATTCTGCCGAGCCTGAGCAGGGATTTTAACGAGCTCATAGCCAATTCGTCAAAAATTTTATTT
ATTACAATTATAATTATTGGGACATTAATTACAGTTACATCTAATTCTTGGTTAGGAGCTTGAATAGGTTTAGAAATTAATTTATTATCTTTTATCCCCC
TATTAAGAGATAATAATAATTTAATATCTACAGAAGCTTCTTTAAAATATTTTTTAACCCAAGTTTTAGCTTCAACTGTTTTATTATTTTCTTCAATTTT
ATTAATATTAAAAAATAATATAAATAATGAAATTAATGAATCTTTTACATCCATAATTATTATATCAGCTTTATTATTAAAAAGTGGAGCCGCTCCTTTC
CATTTTTGATTTCCTAATATAATAGAAGGTTTAACATGAATAAATGCTTTAATATTAATAACTTGACAAAAAATTGCACCTTTAATATTAATTTCTTATC
TCAATATTAAATATTTATTATTAATTAGAGTAATTTTATCGGTTATTATTGGAGCTATTGGAGGACTAAATCAAACTTCTTTACGAAAATTAATAGCATT
TTCTTCAATTAATCATTTAGGATGAATATTAAGATCTTTAATAATTAGAGAATCAATTTGATTAATTTTATTTTTTTTTTTATTCATTTTTATCATTTGTA
TTAACATTTATATTTAATATTTTTAAATTATTTCATTTAAATCAATTATTTTCTTGATTTGTTAATAGAAAAATTTTGAAATTTACATTATTTATAAATT
TTTTATCATTAGGAGGATTACCTCCATTTTTAGGATTTTTACCAAAATGACTTGTAATTCAACAATTAACATTATGTAATCAATATTTTATATTAACAAT
TATAATAATATCAACTTTAATTACATTATTTTTTTATTTACGAATTTGTTATTCCGCTTTTATAATAAATTATTTTGAAAATAATTGAATCATAAAGATA
AATATAAATAGTATTAATTATAATATATATATAATTATAACTTTTTTTTCAATTTTTGGATTATTTTTAATTTCTTTAGATATTGGAACTTTATATTTTA
TTTTTGGAGCTTGAGCTGGAATAGTTGGAACATCTTTAAGAATTTTAATTCGAGCTGAATTAGGACATCCTGGAGCATTAATTGGAGATGATCAAATTTA
TAATGTAATTGTAACTGCACATGCTTTTATTATAATTTTTTTTATGGTTATACCTATTATAATTGGTGGATTTGGAAATTGATTAGTGCCTTTAATATTA
GGTGCTCCTGATATAGCATTCCCACGAATAAATAATATAAGATTTTGACTACTACCTCCTGCTCTTTCTTTACTATTAGTAAGTAGAATAGTTGAAAATG
GAGCTGGAACAGGATGAACTGTTTATCCACCTTTATCCGCTGGAATTGCTCATGGTGGAGCTTCAGTTGATTTAGCTATTTTTTCTCTACATTTAGCAGG
GATTTCTTCAATTTTAGGAGCTGTAAATTTTATTACAACTGTAATTAATATACGATCAACAGGAATTTCATTAGATCGTATACCTTTATTTGTTTGATCA
GTAGTTATTACTGCTTTATTATTATTATTATCACTTCCAGTACTAGCAGGAGCTATTACTATATTATTAACAGATCGAAATTTAAATACATCATTTTTTG
ACCCAGCGGGAGGAGGAGATCCTATTTTATATCAACATTTATTTTGATTTTTTGGTCACCCTGAAGTTTATATTTTAATTTTACCTGGATTTGGAATAAT
TTCTCATATTATTAGACAAGAATCAGGAAAAAAGGAAACTTTTGGTTCTCTAGGAATAATTTATGCTATATTAGCTATTGGATTATTAGGATTTATTGTA
TGAGCTCATCATATATTTACCGTTGGAATAGATGTAGATACTCGAGCTTATTTTACCTCAGCTACTATAATTATTGCAGTTCCTACTGGAATTAAAATTT
TTAGTTGATTAGCTACTTTACATGGAACTCAACTTTCTTATTCTCCAGCTATTTTATGAGCTTTAGGATTTGTTTTTTTATTTACAGTAGGAGGATTAAC
AGGAGTTGTTTTAGCTAATTCATCAGTAGATATTATTTTACATGATACTTATTATGTAGTAGCTCATTTTCATTATGTTTTATCTATAGGAGCTGTATTT
GCTATTATAGCAGGTTTTATTCACTGATACCCCTTATTTACTGGATTAACGTTAAATAATAAATGATTAAAAAGTCATTTCATTATTATATTTATTGGAG
TTAATTTAACATTTTTTCCTCAACATTTTTTAGGATTGGCTGGAATACCTCGACGTTATTCAGATTACCCAGATGCTTACACAACATGAAATATTGTATC
AACTATTGGATCAACTATTTCATTATTAGGAATCTTATTCTTTTTTTTTATTATTTGAGAAAGTTTAGTATCACAACGACAAGTAATTTACCCAATTCAA
CTAAATTCATCAATTGAATGATACCAAAATACTCCGCCAGCTGAACATAGATATTTAATAGAACAATTAATTTTTTTTCATGATCATGCATTATTAATTT
TAGTAATAATTACAGTATTGGTGGGATATTTAATATTTATATTATTTTTTAATAATTATGTAAATCGATTTCTTTTACATGGACAACTTATTGAAATAAT
TTGAACTATTTTACCAGCAATTATTTTACTATTTATTGCTCTTCCTTCTTTACGTTTACTTTATTTATTAGATGAAATTAATGAACCATCTGTAACTTTA
AAAAGAATCGGCCATCAATGATATTGAAGTTACGAATATTCAGATTTTAATAATATTGAATTTGATTCATATATAATTCCAACAAATGAATTAATAACTG
ATGGATTTCGATTATTAGATGTTGATAACCGAGTAGTTTTACCTATAAACTCACAAATTCGAATTTTAGTAACAGCTGCTGATGTTATTCATTCTTGAAC
AGTACCTGCTTTAGGAGTAAAAGTTGACGGTACACCTGGACGATTAAATCAAACTAATTTTTTTATT

D_mojavensis

GCCGATGGCGGCACCTCGGGCACTGGCGGCAGCACGGCCGATCCCGGCTCGAAGAGCTTCCCGGGCGTGCCCTTCTCCTCGCTGGACTTCAATCCGACCT
GTGCGATCACCAACTACGCCGATCCCACCAATGTGCGCAACTGCGAGCTGGTGGGCCTGCGCGACTTGAACCAGGGCAACTCCTATGTGCGCGACAAGAT
CGTGGACTTCCTCAACCATTTGACGGATCTGGGCGTTGCCGGCTTCCGTGTGGACGCCGCCAAGCACATGTGGCCCGGAGATCTGCAGGCCATCTACAGC
CGCCTGAACAATCTGAACACTGCGCACGGCTTCAGCTCGGGCGCCAGGCCCTACATCTTCCAGGAGGTGATTGACTTGGGCAACGAGGCCATCTCCAAGT
CGGAGTACACCGGCCTGGGCGCCGTCACCGAGTTCCGTCACTCGGACTCCATTGGCAAGGTGTTCCGCGGCAAGGATCAGCTGCGCTACCTGAACAACTG
GGGCACCGCCTGGGGCTTCGCCGACTCCGATGCCTCGCTTGTGTTTGTGGACAATCACGACAACCAGCGTGGCCATGGCGCCGGTGGCGCCGATGTGCTC
ACCTACAAGGTGCCCAAGCAGTACAAGATGGCCTCCGCCTTCATGCTGGCCCATCCGTTCGGCACGCCCCGCGTGATGTCCTCCTTTGCCTTCGACAACA
CCGACCAGGGCCCCCCGACCACCGATGGCCACAACATCGCCTCGCCCATCTTCAACAGCGACATGTCCTGCGGCGGTGGCTGGGTGTGCGAGCACCGCTG
GCGCCAGATCTACAACATGGTTGGCTTCCGCAACGCCGTCGGCGACGCCCGCCTCATTTACAGCCACACGCGCAACCTGAACACAGACCACGGCTTCCCC
AACAACGCACGGCCCTTCATCTACCAGGAGGTCATCGATCACGGCCACGAGACGGTCTCGCGGGATGAGTACACGCCCCTGGGAGCCGTCACCGAGTTCC
GTTTCTCAGAGGAAATCGGCAAGGCATTCCGCGGCAACAATGCGCTGAAATGGCTATCGAGCTGGGGCACCGACTGGGGATTCCTGCCCTCTGAGCAGGC
ACTCACGTTCGTGGACAACCACGACAATCAGCGCGACGGCGGCCAGGAGCTGAACTACAAGTCGCCCAAGCAGTACAAGATGGCCACCGCCTTCCATTTG
GCCTATCCCTATGGCATCAGCCAGGTGATGAGCTCCTTTGGCTTCGATGATCGCGACCAGGCGCCACCCCAGGATGCCCAGGAGCGCATCATCTCGCCGG
AGTTCGATGAGGACGGCGGCTGCATGAACGGCGCCTCCGTGGAGATCGTCACCGAAACCTTGGATGTGTACAATGAGTTCATGGATGCCGCCAGGCAGGA
GCACATGTGCCTGATGCACTTCAAGAGCGACGACAACATCTACATACTCTTCGGCAACAAGATGGCCAGTCACTTCAAGGAGAACGGCACGGTGTTTGCC
GTGCCCACGGACCGTGCAGATCAGGTATTCCTAGAGGAGCTGCCTAACAAGGCTTTCGTTCTGATGGAGAATGACATCGAGCTGCGAGCG---
GGTGACCTGGATCCGATGCCCACGGAGCTCGACGAGGTGCTCATTGGCAAGAGTGTGCTGCCCTCGCGCGTCCTAGTCTTCGCCAGCCCCGTCGTTGATC
TGATGAGCTCGATGAGCTACGCGAGCATGGGCTCGCCTCTGGGCAACATG---
GGCGGCTGCATGGCCATGTCGGCGGCGAGCATGTCGGCGGCTGGCTTGGGCGGCAGCTACGGATCGATGCCACCAGGCACGCGGGACATGGAGCCCGGCT
CACCCAACTCCCTGGGACGACGGGGCGTGGACAAGCCAACTACATACAGACGCAGCTACACGCACGCGAAGCCGCCTTACAGCTACATCTCGCTCATCAC
GATGGCCATACAGAACAATCCTACACGCATGCTTACCTTGTCGGAGATCTATCAGTTCATCATGGATCTGTTCCCGTTCTATCGGCAGAATCAACAGCGC

TGGCAGAACTCCATACGTCACTCACTGAGCTTCAACGACTGCTTCGTGAAGATACCACGCACTCCGGACAAGCCGGGCAAGGGCTCCTTCTGGACGCTGC
ACCCGGACTCCGGCAACGACAATCATTGCATCAATGCAGATGTCTTTGTGCTGGTCCTCAATGCTGAGTCGACAATGACGCGGGCCGAGAAGCAGTTCTT
TCACACTGTTTCCCAAAAGCTGTCCAAGCCAAACATCTTCATATTGAACAATCGCTGGGATGCGTCAGCCAATGAGCCCGAGTTTCAAGAATCGGTCAAG
TCGCAGCACACGGAGCGCTGCATCGATTTCCTTACCAAGGAGCTGAAGGTCAGCAACGAGAAGGAGGCTGCGGAGCGCGTGTTCTTCGTGTCGGCGCGCG
AGACGCTGCAGGCGCGCATCGAGGAGGCGAAGGGCAATCCGCCGCATATGGGTGCCATCGCTGAGGGCTTTCAGATACGCTACTTTGAGTTCCAGGACTT
TGAGCGCAAGGTGCTGCCCCCGATATTACAATGCTCGAGCGGGCATTTGGTCTGCGTATCGTGCCGATCAAAGCTCACATGCTGCCCCACATGCCGCGGC
CCGTTGGCCAACATACGCAATCTGGCCATGGAGAAGGTCGCCACCAATGTGAAATTTCCGTGCAAGCATTCGGGCTACGGCTGCACTGCCTCACTTGTTT
ACACAGAGAAGACCGAGCACGAGGAGACCTGCGAATGCCGGCCATACCTATGCCCCTGTCCGGGCGCCTCATGCAAATGGCAGGGACCGCTCGATCTAGT
CATGCAGCATCTGATGATGTCACACAAGAGCATTACCACGCTGCAGGGCGAAGATATTGTCTTTCTGGCCACCGATATCAATCTGCCCGGTGCCGTCGAC
TGGGTTATATTTAAGGAAATTGGCAGTGCCTCCAATGCGCTGCGTACTATGCAGGGCTTTCCATTCTATGACAAACCCATGCGCATTGCCTACTCCAAGT
CCGATTCGGATATTGTGGCCAAATTGAAGGGCACCTACAAGGAGCGTCCAAAGAAGGTGAAGCCGCCAAAGCCGGTGCCGGGCGTCGAGGAGAAAAAGGA
CAAGAAGAAGAAGCAGAGCAGTGCAGAAAATGCAAATCCGAATACACAAACAGAGCAGCCACCAAATCAAATCCTTTTCCTCACCGCGCATGCGGTGCTG
GGCAAGCACGACAATGTGGTTCGCTACTATTCGGCCTGGGCGGAGGACGATCATATGTTGATACAGAATGAATTTTGTGATGGGGGCAGCCTGCATGCGC
GAATCCAGGAGCATTGCTTGGGCGAATCCGAGCTTAAGATCTTGCTGATGCATGTGATCGAGGGCCTGCGTTATATACATTCCAATGATTTGGTGCACAT
GGACATCAAGCCAGAGAACATATTCTCCACCATGAATCCCACTGCGCACAAGAAAGATGATGACGGCATGGATAGCGTGTACGAGGAGCTGCGCAGCTCT
GAGAATCTTGTAACATACAAAATTGGTGACTTAGGACATGTGACGTCCGTGAACGAGCCGTACGTCGAGGAGGGTGATTGCCGCTATCTGTCCAAGGAGA
TACTGCAGGAAGACTATAGCAATCTGTTCAAGGCGGACATCTTCTCGCTGGGCATAACGCTCTTCGAGGTGGCTGGCGGCGGACCATTGCCAAAGAACGG
ACCCGAGTGGCACAAGTTACGCAACGGAGAGGTGCCGGCCATACCAGCGTTGAGCAAGGACTTTAATGAACTCATCGCGAATTCTTCAAAAATTTTATTT
ACTGCAATAATAATTATTGGAACTTTAATTACAGTAACTGCAAATTCTTGGTTAGGAGCTTGAATAGGTTTAGAAATTAATTTACTATCTTTTATCCCCC
TTATAAGAGATAGAAATAATTTAAAATCTACTGAAGCTTCATTAAAATATTTTTTAACCCAAGCTTTAGCTTCTACAGTTTTATTGTTTTCTGTTATTTT
ATTAATATTAAAAAATAACATAAATTACGAAATTAGTAATTCTTTTATTTCTATAATTATTACATCAACTTTATTGTTAAAAAGAGGGTCAGCTCCGTTT
CATTTTTGATTTCCAAATTTAATAGACGGATTAACGTGAATAAATGCTTTACTATTAATAACTTGACAAAAAATTGCACCTTTAATATTAATTTCTTATT
TAAATACAAAAAATATTTTATTAATTAGAGTAATTTTGTCAGTCATTGTAGGGGCTATTGGTGGTTTAAACCAAACATCCCTACGAAAATTAATAGCTTA
TTCTTCTATTAACCATTTAGGATGAATATTAAGAGCATTAATATTAAATGAATCTGTTTGATTAATTTATTTTTTATTTTATTCTTTGCTATCATTTACA
TTAATTTTTATATTTAATATTTTTAAACTATTTCATTTAAATCAGTTATTTTCTTGGTATTCCTGTAATAAAATTTTAAAATTTGTTTTATTCACAAATT
TTTTATCTTTAGGAGGATTACCCCCATTTTTAGGATTTTTGCCAAAATGAATTGTAATTCAACAACTAACTTTTTGTAATCAATATTTTCAATTATTAAT
TTTATTAGTAACAACATTAATTACACTATTTTTTTATCTACGAATATGTTACTCAGCATTTATACTAAATTATTACGAAATCGTTTGAATAAATAATTCA
CAAGTAAACAATTTTACTTTAAATTTATTTATATTACTATCATTTTTTTCAATTTTTGGTTTAATTTTTATTTCAATAGATATTGGAACTTTATATTTTA
TTTTCGGAGCTTGAGCTGGAATAGTGGGAACTTCTTTAAGAATTTTAATTCGTGCTGAATTAGGTCATCCAGGTGCACTAATTGGAGATGATCAAATTTA
TAATGTAATTGTCACAGCACACGCTTTTGTAATAATTTTTTTTATAGTAATACCTATTATAATTGGGGGATTTGGAAATTGACTAGTACCTTTAATACTT
GGGGCCCCTGATATGGCATTCCCTCGAATAAATAATATAAGATTTTGACTTTTACCCCCAGCTTTAACTTTATTATTAGTAAGCAGTATAGTTGAAAACG
GAGCTGGAACAGGGTGAACTGTCTACCCTCCGCTATCTTCAGGTATTGCCCACGGAGGAGCCTCAGTAGATTTAGCAATTTTTTCTTTACATTTAGCAGG
AATTTCTTCTATTTTAGGTGCCGTAAATTTTATTACAACAGTAATTAACATACGATCAACCGGAATTACTCTTGACCGTATGCCTTTATTTGTGTGATCT
GTTGTAATTACAGCTTTATTGCTACTTTTATCCTTACCTGTCTTAGCTGGGGCTATTACAATATTATTAACAGACCGAAATTTAAATACTTCATTTTTTG
ACCCCGCAGGAGGAGGAGACCCAATTCTTTACCAACATTTATTTGATTTTTTGGACACCCAGAAGTATATATTTTGATTTTACCGGGATTTGGAATAAT
TTCTCATATTATTAGTCAAGAATCAGGTAAAAAGGAAACATTTGGTTCTTTAGGAATAATTTATGCTATACTAGCAATTGGTTTACTAGGATTTATTGTA
TGAGCTCACCATATATTTACAGTTGGAATAGATGTAGACACACGAGCTTATTTTACATCAGCAACAATAATTATTGCCGTACCTACTGGGATTAAAATTT
TTAGTTGATTAGCTACTCTTCACGGAACTCAACTTACATATTCCCCAGCTATTTTATGAGCATTAGGATTTGTATTTCTATTTACAGTTGGTGGATTAAC
TGGAGTAGTTTTAGCTAATTCTTCTGTTGATATTATTTTACATGACACTTATTATGTAGTAGCTCATTTTCACTATGTTTTATCAATAGGAGCAGTTTTC
GCAATTATAGCAGGATTTATCCATTGATACCCGTTATTTACAGGATTAACTCTAAATTCTAAATGATTAAAAAGTCAATTTATTATTATATTTATTGGTG
TAAATTTAACATTTTTCCCTCAACATTTTTAGGATTAGCAGGAATGCCTCGACGATACTCAGATTACCCTGATGCTTATACAACATGAAATGTAATTTC
TACTATTGGTTCATCAATTTCATTATTAGGAATTTTATTCTTTTTTACATTATCTGGGAAAGTTTAGTATCTCAACGCCAAGTTATTTATCCAATTCAA
TTAAATTCATCAATTGAATGATACCAAAACACTCCCCCCGCTGAACACAGATACTTAATAGAACAATTAATTTTTTTTCATGACCACGCTTTATTAATTT
TAGTAATAATTACTGTTTTAGTAGGTTATTTAATATTTATACTATTTTTTAACAATTATGTAAATCGATTTTTGTTACATGGTCAATTAATTGAAATAAT
TTGAACTATTTTACCGGCAATTATTTTGTTATTTATTGCTTTCCTTCTTTACGATTATTATACCTTTTAGATGAAATTAATGAACCTTCAGTAACTTTA
AAAAGAATTGGTCACCAGTGATATTGAAGTTATGAATATTCAGATTTTAATAATATTGAATTTGATTCATATATAATCCCTACTAATGAATTATCAACTG
ATGGATTCCGTTTATTAGATGTAGATAACCGAATTGTTCTTCCTATAAATTCTCAAATTCGAATTTTAGTGACAGCCGCAGATGTAATTCATTCATGAAC
AATCCCAGCTTTAGGAGTAAAAGTTGATGGTACTCCAGGACGATTAAATCAAACTAATTTCTTTATA

D_nannoptera

GCCGATGGCGGCAATTCGGGCACGGGCGGCAGCACTGCCGATCCCAGTTCCAAAGACTTCCCCGGTGTGCCATTCTCTTCGCTAGACTTCAATCCGACGT
GCGCGATCACCAACTATGCTGATCCCAGCAATGTGCGTAACTGCGAGCTGGTCGGTCTGCGTGATCTGAACCAGGGCAACTCCTGGGTGCGCGACAAGAT
AGTCGATTTCCTGAACCATTTGACGGACTTGGGTGTGGCCGGTTTCCGTGTGGATGCCGCCAAGCATATGTGGCCCGGAGATTTGGATGCGATCTACAGT
CGTCTCAACAATTTGAATACAGATCATGGCTTCGATTCGGGCGCCAGGCCTTATATCTTCCAGGAGGTGATCGATTTGGGCGGCGAAGCCATTTCCAAGT
CGGAGTACACCGGTATGGGTGCCGTCACCGAGTTCCGTCATTCCGACTCCATTGGCAAGACGTTCCGCGGCAAGGATCAGCTGCGCTATCTGAACAACTG
GGGCACCGCCTGGGGCTTTGCTGCCTCCGATCGCTCGCTGGTCTTTGTGGACAATCATGACAATCAGCGCGGACATGGCGCCGGTGGCGCCGATGTGCTC
ACCTACAAGGTGCCCAAGCAGTACAAGATGGCCTCCGCCTTCATGTTGGCCCATCCCTTCGGCACCCCGCGTGTAATGTCCTCGTTCGCCTTCGACGACA
CGGACCAGGGACCGCCGACCACCGATGGCCACAACATTGCCTCGCCCCAATTCAATGGCGAAAATACCTGCGGCGGCGGTTGGGTGTGCGAGCACCGTG
GCGCCAGATCTACAACATGGTCGCCTTCCGCAATGCCGTGGAGAATGCCGACCTGATTTACAGCCACATGCGCGACCTGAACACGGATCATGGCTTCCCC
AAGAACGCCCGCCCCTTCATCTACCAGGAGGTCATCGATCATGGCCACGAGACAGTTTCGCGCGATGAATACACGCCTCTGGGCGCCGTCACCGAGTTCC
GTTTCTCGGAGGAAATCGGCAAGGCTTTCCGCGGCAACAATGCCCTGAAATGGCTGCAGAGCTGGGGCACCGACTGGGGCTTCCTGCCCTCCGAGCAGGC
GCTCACCTTTGTGGACAACCACGACAACCAGCGCGATGGTGGTCAGGAGCTGAACTACAAGTCTCCCAAGCAATACAAAATGGCCACCGCCTTCCACTTG
GCCTATCCCTACGGCATCAGCCAGGTGATGAGCTCGTTTGGCTTCGATGATCGCGACCAGGCGCCGCCACAGGACGCCCAGGAGCGTATCATCTCCCCGG
AGTTCGATGAAGACGGCGGTTGCACCAACGGCGCCTCAGTGGAGATAGTCACGGAAACGCTCGACATCTACAATGAGTTCATGGACGCGGCCAGAGAGGA
GCACATGTGCCTCATGCACTTCAAGAGCGATGACAACGTGTACATACTCTTCGGCAACAGGATGGCCAATCACTTCAGGGAGAACGGCACGGTGTTTGCC
GTGCCCATGGAACGGGCGGATCAGGTGTTCCTGGAGGAGCTGCCCAACAAGGCGTTCATACTGATGGAGAACGACATCGAGCTGCGAGCG---
GCTGATCTGGATCCGATGCCCACGGCCCTGGACGAGGTCCTCATCGGCAAGAGTGTGCTGCCCTCGCGAGTCCTGGCCTTCGCTAGGCCTGTCGTGGACC
TGATGAGCTCGATGAGCTACGCGAGCATGGGCTCGCCGCTGGGCAACATG---
GGCGGCTGCATGGCCATGTCGGCGGCGAGCATGTCGGCGGCGGGCTTGGGCGGCAGCTACGGTTCGATGCCACCGGGCACGCGAGACATGGAGCCGGGCT
CACCGAATTCCCTCGGCCGTCGCGGCGTCGACAAGCCAACCACATACAGACGCAGCTACACGCACGCGAAGCCTCCGTACAGCTACATCTCGCTCATCAC
GATGGCCATACAGAACAATCCGACGCGCATGCTGACGCTCTCCGAGATCTATCAGTTCATCATGGATCTGTTCCCGTTCTACAGACAGAACCAGCAGCGC
TGGCAGAACTCCATACGCCACTCGTTGAGCTTCAACGATTGCTTCGTGAAGATACCGCGGACGCCGGACAAGCCCGGCAAGGGCTCGTTCTGGACGCTGC
ATCCGGACTCGGGCAATGATAATCATTGCCTCAACGCAGATGTCTTCGTGTTGGTCCTTAACGCGGAGTCCACAATGACGCGGGCCGAGAAACAATTCTT
TCACACCGTTTCACAGAAACTATCCAAACCGAATATCTTCATACTGAACAACCGCTGGGACGCTTCGGCCAATGAGCCAGAGTTCCAAGAATCGGTGAAG
TCCCAGCATACGGAACGCTGCGTGGATTTTCTCACCAAAGAGCTGAAGGTCAGCAACGAGAAAGAGGCCGCTGAGCGTGTCTTCTTTGTCTCCGCACGTG
AGACGCTGCAGGCTCGGATCGAGGAGTCCAAGGGCAATCCGCCACATTTGGGCGCCATCGCTGATGGATTTCAGATACGCTACTTTGAGTTTCAGGACTT
TGAGCGTAATGTGCTGCCACCGATATTGCAATGCTCTAGCGGGCACTTGGTGTGCGTTTCGTGCCGCTCAAAGCTCACATGCTGCCCAACATGCCGCGGC
CCATTGGCCAATATACGTAACCTGGCTATGGAGAAGGTCGCGTCGAATGTGAAGTTCCCGTGCAAGCATTCGGGCTACGGCTGCACTGCCTCACTAGTTT
ACACAGAGAAGACCGAGCACGAGGAGACCTGCGAATGCCGGCCATACCTATGCCCCTGCCCGGGCGCCTCATGCAAATGGCAGGGTCCGCTCGATCTAGT
CATGCAGCATCTGATGATGTCCCATAAGAGTATCACAACGCTGCAAGGCGAAGATATTGTATTTCTGGCCACCGATATAAATTTACCCGGCGCCGTTGAC

TGGGTTATATTTAAGGAAATTGGCAGCGCCTCTAATGCTCTGCGCACCATGCAGGGCTTTCCATTCTATGACAAACCAATGCGTATTGCCTATTCCAAAA
CGGACTCGGATATTGTTGCCAAATTAAAGGGCACGTATAAAGAACGACCAAAGAAGGTGAAACCGGTGAAGCCGGCACTGGGCGCTGAAGAGAAAAAGGA
CAAGAAGAAGAAGCAAAGCAGCGCCGAGAATTCCAACTCGAACGCACAAACAGAACAGCCACCAAATCAAATTCTCTTCCTAACCGCGCATGCGGTCTTA
GGCAAGCACGACAATGTCGTGCGCTACTATTCTGCTTGGGCGGAGGATGATCATATGTTGATCCAAAACGAATTTTGTGATGGCGGCAGTCTGCATGCTC
GAATCCAGGAGCACTGTTTAGGGGAATCCGAGCTTAAGATATTGTTAATGCATGTGATCGAAGGTTTGCGTTATATACATTCTAATGATTTGGTGCACAT
GGACATCAAACCAGAGAACATTTTCTACACAATGAATCCAACGGCTCACAAGAAAGACGATGATGGGATGGATAGTGTATATGAGGAGTTGCGCAGCTCC
GAGAACCTCGTCACATATAAGATTGGGGACTTAGGACACGTTACATCTGTCAATGAGCCGCACGTCGAGGAAGGAGACTGTCGCTATCTGCCCAAGGAAA
TTTTGCAGGAAGACTACAGTAATCTCTTCAAGGCGGATATCTTTTCGCTGGGTATTACTCTATTCGAGGTAGCAGGCGGTGGTCCTTTGCCGAAGAATGG
ACCCGAATGGCACAAGCTGCGCAGTGGAGAGGTACCACCTATTCCTACGCTAAGCAAGGACTTTAACGAGTTGATTGCAAATTCTTCAAAAATTTTATTT
TTAACAATAATAATAATTGGAACTTTAATTACAATTACATCTAATTCTTGATTAGCCGCTTGAATAGGATTAGAAATTAATTTATTATCTTTTATCCCCC
TTATAAGAGATGTAAATAATTTAAAATCCACAGAAGCTGCTTTAAAATATTTTTTAACTCAAGCTTTAGCTTCAATTGTATTGTTATTTTCCGTTATTTT
ATTAATATTAAAAATTAATTTAAATTACGAAATTAATTTATCGTTTATTTCCGTAATAATTTTATCAACTTTATTACTAAAAAGAGGAGCAGCTCCTTTC
CACTTTTGATTCCCTAATTTAATAGAAGGTTTAACTTGAATAAATTCTCTTTTATTAATGACATGACAAAAAATAGCACCGTTAATATTAATTTCTTATT
TAAATTTAAAAAATTTTTTACTAGTTAGTGTAATTTTATCTGTGATTATTGGAGCAATCGGAGGCTTAAACCAAACTTCTTTACGAAAATTAATAGCTTT
TTCTTCAATTAATCATTTAGGATGAATATTAAGTGCTTTATCAATTAATGAAATAAATTGATTAATTTATTTTTTATTTTATTCATTTTTATCATTAACA
TTAACATTAATATTCAATAATTTTAAATTATTTCATTTAAATCAATTATTTTCTTGATTTATTCAAAATAAAATTTTAAAATTTACTTTATTTATAAATT
TTTTATCTTTAGGTGGACTTCCACCATTTTTAGGATTTATGCCTAAATGAATTATTATTCAGCAATTAACATTTTCAAATCAATATTTTGAATTATTTAT
TTTATTAATATCAACATTAATTACTCTATTTTTTTATTTGCGAATTTGTTATTCAGCGTTTATGCTTAATTATTATGAAACTAATTGATTAATAAATCAA
TTTTTAAATAATATTTCAATAAATTTTTATCTAATTTTAAGATTTTTTTCTATTTTTGGATTATTTTTTATTCCTATGGATATCGGAACATTATATTTTA
TTTTTGGTGCTTGAGCAGGAATAGTTGGGACATCTTTAAGAATTTTAATTCGGGCGGAATTAGGTCACCCAGGAGCTTTAATTGGAGATGACCAAATTTA
TAATGTAATTGTTACAGCTCATGCTTTCGTTATAATTTTTTTTATAGTAATACCAATTATAATCGGGGGATTCGGAAATTGATTAGTTCCTTTAATATTA
GGAGCCCCAGATATAGCTTTCCCACGAATAAATAATATAAGATTTTGATTATTACCTCCAGCATTAACACTTTTATTGGTTAGAAGAATAGTTGAAAATG
GAGCTGGGACAGGATGAACAGTGTACCCCCCTCTTTCAGCAGGAATCGCTCATGGCGGGGCTTCTGTAAATTTAGCAATTTTTTCTCTTCATTTAGCCGG
AATTTCTTCAATTTTAGGGGCTGTAAATTTTATTACAACAGTAATTAATATGCGATCTGCAGGAATCACTCTTGACCGAATACCTTTATTTGTTTGATCT
GTAGTAATTACAGCCTTACTTTTATTACTTTCATTACCAGTTTTAGCCGGAGCTATTACTATATTATTAACAGATCGAAATTTAAATACTTCTTTTTTTG
ACCCTGCAGGAGGAGGAGATCCTATTTTATACCAACATTTATTTTGATTTTTTGGACACCCAGAAGTATACATTCTTATTTTACCTGGATTTGGAATAAT
TTCTCACATTATTAGCCAAGAATCGGGGAAAAAAGAAACTTTTGGATCATTAGGAATAATTTATGCTATATTAGCTATTGGTTTATTAGGGTTTATTGTT
TGAGCTCATCATATATTTACCGTAGGTATAGATGTGGATACTCGAGCTTATTTTACATCCGCAACTATAATTATTGCTGTACCAACAGGAATTAAAATTT
TTAGATGATTAGCTACACTTCATGGTACTCAACTTTCTTATTCCCCGGCTATTTTATGAGCTTTAGGTTTTGTATTTTTATTTACAGTCGGAGGATTGAC
CGGAGTTGTTTTAGCAAATTCTTCTATTGATATTATTCTTCACGAAACATATTATGTTGTAGCTCATTTTCATTATGTACTATCTATGGGAGCTGTATTC
GCTATTATAGCAGGATTTATTCATTGATACCCTTTATTAACAGGATTAACTTTAAATAATAAATGATTAAAAAGTCAATTTATTATTATATTTATTGGAG
TTAATTTAACTTTTTTCCCTCAACATTTTTTAGGGTTAGCGGGGATACCACGTCGATATTTTGATTACCCTGATGCTTACACAACATGAAATGTAATTTC
AACTATTGGATCATCTATTTCTCTTTTGGGAATTTTATTCTTTTTTTTTATTATTTGAGAAAGTTTAATTTCTCAACGACAAGTAATTTATCCAATTCAA
TTAAATTCTTCAATTGAATGATATCAAAATACACCCCCAGCAGAACATAGTTATTTAATAGAACAATTAATTTTTTTTCATGACCACGCATTGTTAATTT
TAGTTATAATTACAGTTCTTGTAGGTTATTTAATATTAACTTTATTTTTTAATAAATATAATAATCGATTTTTATTGCACGGACAATTAATTGAAGTAAT
TTGAACAATTTTACCCGCTATTATTTTACTTTTTATTGCATTTCCTTCTTTACGATTATTATATCTTATTGATGAAATTAATGAACCTTCTGTAACTTTA
AAAAGAATTGGGCATCAATGGTATTGAAGCTATGAATATTCTGATTTTAATAATATCGAATTTGATTCTTATATAATTCCTACAAACGAATTAAATAATG
ATGGATTTCGTTTATTAGATGTTGATAACCGAATTATTTTACCTATAAACTCCCAAATTCGAATTTTAGTAACAGCCGCTGATGTAATTCATTCATGAAC
AATTCCTGCTTTAGGAGTAAAAGTTGATGGAACTCCAGGTCGATTAAATCAAACTAATTTTTTTATT

D_pachea

GCCAATGGCGGCAATTCGGGCACTGGCGGCAGCACTGCCGATCCTGGATCGAAGGACTTCCCCGGTGTGCCATTCTCCTCGCTGGACTTCAATCCTACGT
GCGCGATTACCAACTATGCTGATCCCAGCAATGTGCGTAACTGCGAACTGGTTGGCCTGCGCGATCTGAACCAGGGCAACTCCTGGGTGCGTGACAAGAT
CGTCGATTTCCTGAACCACTTGACCGACTTGGGTGTGGCCGGTTTCCGTGTGGATGCCGCCAAGCATATGTGGCCCGGAGATTTGGATGCGATCTTCAGT
CGTCTGAACAATTTGAATACAGATCATGGCTTCGATTCGGGCGCCAGGCCGTTCATCTTCCAGGAGGTGATCGATCTGGGCGGCGAAGCCATCTCCAAGT
CCGAATATACCGGCATGGGTGCCGTCACTGAGTTCCGTCATTCGGACTCCATTGGCAAGGTATTCCGCGGCAAGGATCAGCTGCGCTATCTGAACAACTG
GGGCACCGACTGGGGCTTTGCGCCCTCCGATCGCTCTCTGGTCTTTGTGGACAATCATGACAATCAGCGTGGACATGGCGCCGGTGGCGCTGATGTGCTC
ACCTATAAGGTGCCCAAGCAATACAAGATGGCATCCGCCTTCATGTTGGCCCATCCCTTCGGCACGCCGCGTGTGATGTCCTCGTTCGCCTTCGATGACA
CCGACCAGGGACCACCGACCACCGATGGCCATAACATCGCATCGCCCCAATTCAACGGCGACAACTCCTGCAGCGGTGGCTGGGTGTGCGAGCACCGTTG
GCGCCAGATCTACAACATGGTCGCCTTCCGCAATGCCGTGGAGAATGCCGACATGATTTACAGCCACATGCGCGACCTGAACACGGCCCATGGTTTCCCC
AAGAACGCACGGCCGTTCATCTACCAGGAGGTGATCGATCATGGCCACGAGACTGTTTCGCGTGATGAATACACGCCTCTGGGCGCGGTCACCGAGTTCC
GTTTCTCCGAGGAAATCGGCAAGGCTTTCCGCGGCAACAATGCACTGAAATGGCTGCAGAGCTGGGGCACCGACTGGGGCTTCCTGCCCTCCGAGCAGGC
GCTCACGTTTGTGGACAACCACGACAACCAGCGCGATGGTGGTCAGGAGCTGAACTACAAGTCTCCCAAGCAGTACAAAATGGCCACCGCCTTCCACTTG
GCCTATCCCTATGGCATCAGCCAGGTGATGAGTTCGTTTGGCTTCGATGATCGCGACCAGGCACCGCCACAGGACGCCCAGGAGCGCATCATCTCTCCGG
AGTTCGATGAAGACGGCGGCTGCATCAATGGCGCCTCTGTGGAGATAGTCACTGAAACCCTGGATATCTACAATGAGTTCATGGACGCGGCCAGAGAAGA
GCACATGTGTCTCATGCACTTCAAGAGCGATGATAATGTGTACATACTCTTTGGCAACAGGATGGCCAATCACTTCAAGGAGAACGGCACGGTGTTTGCC
GTGCCCATGGAACGGGCGGATCAGGTTTTTCTGGAGGAGCTGCCCAACAAGGCATTCATACTGATGGAGAACGACATCGAACTGCGAACT---
GCTGATCTGGATCCTATGCCCACGGCCCTGGATGAGGTTCTCATTGGCAAGAGTGTGCTGCCCTCGCGAGTCCTGGTCTTCGCACGGCCTGTCGTGGATC
TGATGAGTTCGATGAGCTATGCGAGCATGGGCTCACCGCTGGGCAACATG---
GGCGGCTGCATGGCCATGTCCGCGGCAAGCATGTCGGCGGCGGGCTTGGGCGGCAGCTATGGCTCGATGCCGCCGGGCACGCGCGACATGGAGCCGGGCT
CACCGAATTCGCTCGGACGTCGCGGGGTCGACAAGCCGACCACATACAGACGCAGCTACACGCATGCGAAGCCGCCTTACAGCTACATCTCGCTCATCAC
GATGGCCATACAGAACAATCCGACGCGCATGCTGACGCTCTCCGAGATCTATCAGTTCATCATGGATCTGTTCCCGTTCTACAGGCAGAACCAGCAGCGC
TGGCAGAACTCCATACGCCATTCGTTGAGCTTCAACGATTGCTTTGTGAAGATACCCCGGACGCCGGACAAGCCCGGCAAGGGCTCGTTCTGGACGCTGC
ATCCGGACTCGGGCAATGATAATCATTGCATCAACGCAGACGTCTTTGTTTTAGTCCTAAACGCCGAATCCACAATGACACGTGCCGAGAAGCACTTCTT
TCACACCGTTTCACAGAAACTGTCCAAACCGAATATCTTCATACTAAACAATCGCTGGGATGCCTCGGCCAATGAGCCAGAGTTTCAAGAATCGGTTAAG
TCCCAGCATACGGAACGCTGTGTGGATTTTCTCACCAAAGAGCTGAAGGTCAGCAACGAGAAGGAGGCCGCTGAGCGTGTCTTCTTTGTTTCCGCACGTG
AGACGCTGCAGGCTCGGATCGAGGAGTCCAAGGGCAATCCGCCACATTTGGGCGCCATTGCGGATGGATTTCAGATACGCTACTTTGAGTTTCAGGACTT
TGAGCGCAAAGTATTGCCACCGATATTGCAATGCTCCAGCGGGCACTTGGTGTGCGTTTCGTGCCGCTCAAAGCTCACATGCTGCCCAACATGCCGCGGC
CCATTGGCCAATATACGTAACCTGGCCATGGAAAAGGTCGCATCTAATGTGAAGTTCCCGTGCAAGCATTCGGGCTATGGCTGCACTGCCTCACTAATTT
ACACAGAGAAGACCGAGCACGAGGAGACCTGCGAATGTCGGCCATACCTATGCCCCTGCCCGGGCGCCTCATGCAAATGGCAGGGACCGCTCGATCTAGT
CATGCAGCATCTGATGATGTCCCATAAGAGTATCACAACGCTACAAGGTGAAGATATTGTATTTCTGGCCACCGATATTAATTTGCCCGGCGCCGTTGAT
TGGGTCATATTTAAGGAAATTGGCAGCGCCTCTAATGCACTGCGCACCATGCAGGGCTTTCCATTCTATGACAAACCAATGCGTATTGCCTATTCCAAAT
CTGATTCAGATATTGTTGCCAAATTGAAGGGCACGTACAAAGAACGACCAAAGAAAGTGAAACCGCCAAAGCCGGCTATGGGCGCTGATGAGAAAAAGGA
CAAGAAGAAGAAGCAGAGCAGCGCCGAAAATTCAAACCCGAACACACAAACAGAACAGCCACCAAATCAAATTCTCTTCCTTACCGCACATGCGGTATTA
GGCAAGCACGACAATGTCGTGCGCTATTATTCTGCTTGGGCGGAGGATGATCATATGTTGATCCAAAATGAATTTTGTGATGGTGGCAGTCTGCATGCAC
GCATCCAGGAACACTGTTTAGGTGAATCCGAGCTAAAGATATTGCTAATGCATGTGATCGAAGGTTTGCGTTATATACATTCCAATGATTTGGTGCACAT
GGACATCAAACCAGAGAACATTTTCTCCACAATGAATCCAACGGCCCACAAGAAAGACGATGATGGGATGGATAGTGTATATGAGGAGTTGCGCAGCTCT
GAGAACCTTGTCACGTATAAAATTGGAGACTTAGGACATGTTACATCTGTCAATGAGCCGTACGTCGAGGAAGGAGATTGTCGCTACCTGCCCAAGGAAA
TTCTGCAGGAAGACTACAGCAATCTCTTCAAGGCGGATATCTTTTCGCTAGGTATTACGCTATTCGAGGTAGCAGGCGGTGGTCCGTTGCCAAAGAATGG
ACCTGATTGGCATAAGTTGCGCAGTGGAGAGGTACCTCATATCCCCACGCTAAGCAAGGATTTTAACGAGTTGATTGCAAATTCTTCAAAAATTTTATTT

TTAACGATAATAATTATAGGAACATTAATTACAATTTCATCTAATTCTTGGTTAGGAGCTTGAATAGGTTTAGAAATTAATTTGTTATCTTTTATCCCCC
TTATAAGAGATACAAATAATTTAAAATCTACAGAAGCTGCTTTAAAATATTTTTTAACCCAAGCTTTAGCTTCAATTGTACTCTTATTTTCTGTGATTTT
ATTAATATTAAAAATTAATTTAAATTATGAAATTAATTTATCATTTATTTCAATAATAATTTTATCAACTTTGTTATTAAAAAGTGGAGCAGCTCCATTT
CACTTTTGATTCCCTAATTTAATAGAAGGTTTAACTTGAATAAATTCTCTTTTATTAATAACATGACAAAAAATAGCCCCACTAATATTAATCTCTTATT
TAAATTTAAAAAATATGTTAATAATTAGTGTAATTTTATCCGTAATTGTTGGGGCATTTGGAGGTTTAAACCAAACTTCTTTACGGAAATTAATAGCTTT
TTCTTCTATTAATCATTTAGGTTGAATGTTAAGTGCTTTATCTATTAATGAGATATCTTGATTAATTTATTTTTTATTTTATTCATTTTTAACTTTAACA
TTAACATTTATGTTTAATAATTTTAAGTTATTTCATTTAAATCAATTATTTTCTTGATTTGTTAAAAATAAAATTTTTAAATTTACATTATTTATAAATT
TTTTATCTTTAGGAGGACTACCTCCATTTTTAGGATTTTTACCTAAATGAATTGTTATTCAACAATTAACATTTTCAAATCAATATTTTCAATTATTTAT
CCTAATTATATCTACTTTAATTACATTATTTTTTTACCTACGAATTTGTTATTCAGCATTTATATTAAACTATTATGAAAATAGATGAATAATTAATCAA
TTTATTAGTAATATTTCAATAAATTTTTATTTAATTTTATCATTTTTTTCTATTTTCGGATTATTTTTAATTTCTATAGATATTGGAACATTATATTTTA
TTTTTGGTGCATGAGCTGGAATAGTAGGTACATCATTAAGAATTTTGATTCGAGCAGAATTAGGACATCCAGGAGCTTTAATTGGTGATGATCAAATTTA
TAATGTAATTGTTACAGCCCATGCTTTTATTATAATTTTTTTTATAGTTATACCTATTATAATTGGAGGGTTTGGAAATTGACTTGTTCCTTTAATATTA
GGGGCTCCAGATATAGCCTTCCCTCGAATAAATAATATAAGATTTTGACTTCTACCCCCAGCATTAACACTTTTATTAGTAAGTAGTATAGTTGAAAATG
GAGCTGGTACAGGATGAACTGTTTACCCCCCTCTTTCAGCTGGAATTGCTCATGGAGGAGCTTCTGTAGATTTAGCAATTTTCTCTTTACATTTAGCCGG
GATTTCCTCAATTTTAGGGGCTGTAAATTTCATTACAACAGTTATTAATATACGATCAACAGGAATCACACTTGACCGAATACCTTTATTTGTTTGATCT
GTTGTAATTACTGCTTTACTTTTATTACTATCATTACCAGTCTTAGCTGGGGCAATTACAATATTATTAACAGACCGAAATTTAAATACTTCTTTTTTTG
ACCCTGCTGGAGGTGGAGACCCAATTTTATACCAACATTTATTTTGATTTTTTGGACACCCTGAAGTATATATTTTAATTTTACCTGGATTTGGAATAAT
TTCTCATATTATTAGTCAAGAATCAGGTAAAAAGGAAACATTTGGTTCATTAGGTATAATTTATGCTATATTAGCTATTGGTTTATTAGGATTTATTGTA
TGAGCTCATCATATATTTACAGTAGGTATAGATGTTGATACCCGAGCTTATTTTACATCAGCCACTATAATTATTGCTGTCCCAACAGGAATCAAAATTT
TTAGATGATTGGCTACTCTTCACGGAGCTCAACTTTCTTATTCCCCAGCTATTTTATGAGCTTTAGGATTTGTTTTCTTATTTACCGTAGGAGGTTTAAC
TGGAGTTGTCTTAGCTAATTCTTCTATTGATATTATTCTTCATGATACATATTATGTTGTAGCCCATTTTCATTATGTTTTATCAATAGGAGCAGTATTT
GCTATTATAGCAGGATTTATTCATTGATACCCTTTATTTACAGGATTAACTTTAAATAATAAATGATTAAAAAGTCAATTCATTATTATATATTTATTGGAG
TAAATTTAACTTTTTTCCCTCAACATTTCTTAGGATTAGCAGGAATACCTCGACGWTATTCTGATTACCCTGATGCTTACACAACATGAAATGTAATTTC
GACTATCGGATCATCTATCTCTCTATTAGGAATTTTATTTTTTTTCTATATTATTTGAGAAAGTTTAGTTTCTCAACGACAAGTAATTTATCCAATTCAA
TTAAATTCTTCAATTGAATGATACCAAAATACTCCCCCTGCTGAACATAGTTATTTAATAGAACAATTAATTTTTTTCCATGATCATGCATTATTAATTT
TAGTTATAATTACAGTTCTTGTTGGTTATTTAATATTAACTTTATTTTTTAATAAATATATTAATCGATTTTTATTACATGGCCAATTAATTGAAGTAAT
TTGAACAATTTTACCTGCTATTATTTTACTTTTTATTGCATTTCCATCTTTACGTTTATTATACCTTATTGATGAAATTAATGAACCTTCAATAACTTTA
AAAAGAATTGGTCATCAATGATACTGAAGTTATGAATATTCTGATTTTAATAATATTGAATTTGATTCTTATATAATTCCAACAAATGAACTTAGTAGTG
ACGGATTTCGTCTTTTAGATGTTGATAATCGAGTTGTTTTACCTATAAATTCTCAAATTCGAATTTTAGTAACAGCCGCTGATGTAATTCACTCATGAAC
AATCCCAGCTTTAGGAGTAAAAGTTGATGGTACTCCTGGTCGTTTAAATCAAACTAATTTTTTTATT

D_willistoni
GCTGATGGTGGTACTTATGGTACTGCTGGCAGTACTGCCGACCCCAGCACCAAGAGCTTCCCCGGCGTGCCCTTCTCCTCCCTGGACTTCAACCCAACTT
GCTCCATCTCCAACTACAACGATGCCAACCAAGTGCGCAACTGTGAGTTGGTTGGTCTGCGTGATTTGAACCAGGGCAACTCCTGGGTCCAGGACAAGGT
TGTTGAGTTCCTTAACCACTTGATCGAATTGGGTGTTGCTGGATTCCGTGTGGATGCTGCCAAACATATGTGGCCCTCTGACTTGGGTGTCATCTATGGA
CGTCTGAACAACTTGAACACTGCTCATGGTTTCAGCTCGGGTGCCAAGCCTTACATCTTCCAGGAGGTCATCGATATGGGCGGCGAGGCTATTAGCAAGA
CTGAGTACACTGGCATTGGTGCCGTTACCGAGTTCCGTCACTCCGACTCCATTGGCAAGGCTTTCCGCGGCAAGGATCAATTGCAATATCTGGTTAACTG
GGGCACCGCCTGGGGCTTCGCTGCTTCCGATCGCTCTCTGGTCTTTGTTGACAATCATGACAACCAGCGTGGACATGGAGCCGGTGGTGCTGACGTTCTT
ACCTACAAGGTGCCCAAGCAGTACAAGATGGCTTCTGCCTTCATGTTGGCCCATCCCTTCGGCGTTCCTCGTGTGATGTCCTCCTTCTCCTTCGACGATA
CCGACCAGGGACCACCCACTACTGATGGAACCAACATTGCCTCCCCCGGATTCAACAGCGACATGTCTTGCAGCGGTGGATGGGTATGTGAGCACCGTTG
GCGCCAGATCTACAACATGGTTGCCTTCCGCAACGCCGCCGATGGCGCTGAATTGATATACAGTAGCGTGCGTGATTTGAACATTAATCACGGTTTTCCC
AACAATGCCCGCCCCTTTATATATCAAGAGGTCATCGATCATGGCCACGAGACTGTGACTCGCGAGGAATACAACGAGCTGGGCGCAGTCACCGAGTTCC
GTTTCTCGGAGGAAATTGGCAAGGCATTCCGTGGCAACAATGCCCTTAAATGGCTACAGAGTTGGGGCACCGATTGGGGCTTTTTATCTTCCGGTCAGGC
ACTTACCTTTGTGGATAACCATGACAATCAGCGTGACAGTGGAGATGTTCTCAACTACAAATCACCGAAGCAATATAAAATGGCCACTGCTTTCCATTTG
GCCTATCCATATGGCATCAGTCGTGTCATGAGCTCATTCGGCTTCGATGACCGCGATCAGGCACCACCACAGGATGCTCAAGAGCAACTGATTTCTCCTG
AATTCGATGTTGATGGCGGTTGTACCAATGGAGCATCGGTGGAAATAGTCACAGAGAATCTGGAGATGTATAATGATTTCATGGAGGCGGCCCGCCAGGA
GCACATGTGTCTGATGCACTTTAAGAGTGATGAGAATGTTTATATATTGTTTGGCAATAAAATGGCCAATCATTTCAAGGAGAATGGCACCATTTTTTCA
GTGCCCACCGAGCGAACAGACGAAGAGTTTTTAGCAGAATTACCCAATCGTGCCTTTGTTTTGATGGAAAACGAAATTGAACTTCGCACCACCGATCAAC
TGGATCCCACACCCACCAGCCTGGATGAGTCTCTGATAGGCAAGAGTGTTGTGCCCTCACGTATTCTGGCCTTTGCCAGCCCCATAGTGGATCTGATGAG
TTCAATGAGCTATGCCAGCATGGGATCACCATTGGGCAATATAAATGCCAATTGCATGGCAATGTCGGCA---------------------
ATGGGTGGTTCATATGGCTCCATGCCACCGGGCTCACGGGAAATGGAAACGGGTTCACCCAATTCACTGGGCAGATCGCGTGTGGACAAACCGACCACAT
ATCGGCGGAGTTATACACATGCCAAGCCACCATATAGCTACATTTCCCTTATCACCATGGCCATACAGAACAATCCGACGCGAATGCTAACATTGTCGGA
GATCTATCAGTTCATTATGGATCTGTTTCCGTTCTATAGACAAAATCAACAGCGCTGGCAAAATTCCATTAGGCATTCGCTGAGCTTCAACGATTGCTTT
GTCAAGATTCCACGCACACCCGATAAGCCGGGCAAGGGCTCATTCTGGACACTGCATCCGGATTCGGGTAATGACAATCATTGCATTAATGCCGATGTAT
TTGTTCTTGTCCTTAATGCTGAATCCACCATGACCAGAGCAGAAAAGCAATTCTTTCACACGGTTTCACAGAAGCTAAGCAAACCGAATATCTTTATATT
GAACAATCGCTGGGATGCCTCGGCCAATGAACCAGAAAGCCAAGAGTCGGTTAAATCTCAGCATACAGAACGTTGCATTGACTTTCTGACCAAGGAACTG
AAAGTGACCAACGAAAAGGAGGCAGCGGAACGTGTTTTCTTTGTATCGGCACGCGAGACTCTGCAAGCACGCATCGAAGAGTCCAAGGGTAATCCACCAC
ATATGGGTGCCATAGCCGAAGGCTTCCAGATACGTTATTTCGAGTTCCAGGACTTTGAGCGTAAAGTCTTGCCGCCGATATTGCAATGTTCTAGCGGACA
TTTGGTCTGTGTGTCGTGCCGTTCCAAGCTCACCTGCTGCCCCACATGTCGCGGCCCATTGGCCAATATACGAAATTTGGCTATGGAGAAGGTGGCATCA
AATGTAAAATTTCCATGCAAGCATTCGGGCTATGGATGTACTGCCTCTCTGGTATATACAGAAAAAACAGAGCATGAGGAGACTTGCGAGTGTCGTCCAT
ATTTGTGCCCCTGTCCGGGGGCCTCATGCAAGTGGCAAGGACCGCTGGATCTAGTCATGCAGCATCTGATGATGTCGCACAAGAGCATCACAACACTACA
GGGCGAGGATATTGTATTTCTGGCCACCGATATTAATCTTCCCGGTGCTGTCGATTGGGTAATATTTAAGGAAATTGGCAGCGCTTCAAATGCCCTTCGG
ACAATGCAGGGTTTCCCCTTCTATGACAAACCCATGCGCATTGCATACTCCAAATCCGATTCGGATATTGTGGCCAAGCTGAAGGGCACATTCAAGGAGC
GTCCCAAGAAGGTGAAAGTTACAAAGGTGGTGCCTGGAACAGATGACAAAAAGGACAAAAGAAAGAAGCCGGCTAGTACAGAAAATGCAGCTCCCAGCAC
ACAAACCGAGCAGCCGCCAAATCAAATTCTATTCCTTACTGCACATGCTGTTCTGGGCAAGCATGACAATGTGGTACGTTATTATTCAGCCTGGGCAGAA
GATGACCATATGCTGATACAGAATGAATATTGCGACGGCGGCAGTCTGCAATCACGCATCCAGCAGCATCCGCTGGGTGAATCCGAGCTAAAAATTCTAC
TAATGCATGTCATTGAGGGCCTACGCTATATACACTCAAATGATTTGGTTCACATGGATCTTAAGCCAGAAAATATTTTCTCCACCATGAATCCAATGGC
CCACAAAAAAGATGGCGATGGCATGGATAGTGTCTATGAAGAATTGCGTAATTCTGAAAATTTGGTTACATACAAAATCGGTGATTTGGGTCATGTGACC
TCGGTGAAGGAACCGCATGTCGAAGAGGGCGATTGTCGTTATTTACCCAAAGAAATATTGCATGAGGACTATGCGAGTCTATTCAAGGCTGACATATTCT
CATTGGGCATCACCCTCTATGAGGTGGCCGGAGGCGGACCGTTGCCCAAGAATGGCCCAGAATGGCATAAACTAAGAGATGGTGATGTTCCATCATTACC
AGCCTTAAGCAAAGATTTCAATGAATTAATTGCCAATWCTTCAAAAATTTTATTTATCTCAATTATAATTATTGGAACATTAATCACAGTAACTTCTWAY
TCTTGGTTAGGAKCTTGAATAGGTTTAGAAATTAAYTTGTTATCTTTTATCCCCCTAATAAGAGATAATAATAATTTAGTATCTACTGARGCTTCTTTAA
AATATTTTTTAACCCAAGCGTTAGCGTCAACARTTTTATTATTTTCTATTATTTTATTAATATTAAAAAATAATGTAAACARTGAAATTAATGAATYTTT
TACATCTATAATTATAATATCAGCCTTATTAATAAAAAKAGKGGCCGCTCCTTTTCATTTTTGATTTCCTAATATAATAGACGGATTAACATGAATAAAT
GCCTTATTACTAATAACTTGRCAAAAAATTGCACCYTTAATATTAATTTCTTATTTAAATATTAAAAATCTTTTATTAGTAGTGTWATTCTTTCTGTTA
TTGTTGGAGCTATTGGAGGATTAAATCWAACWTCTTTACGAAARTTAATAGCYTTTTCTTCCATTAATCATTTAGGTTGAATATTAAGYGCATTAATAAT
AAATAAATCAATTTGATTAACTTMTTTTTCYTTTTATTTTWTTCTTTCAWTTGTATTAACTTTTATATTTAATYTATTAAAATTATTTCATTTAAATCAA
CTATTTTCAAGATTTTTTAATAGAAAAATTTTAAAATTTACTTTATTTATAAATTTTTTATCTTTAGGAGGATTACCTCCTTTTTTAGGATTTTTACCTA
AATGAATTGTAAWTCAAGAACTAACTTTTTGTAAYCAATACGCTYAATTAATAATTATAATAATRATAACATTAATTACTTTATTTTTTTATTTACGAAT
TTGTTACTCAGCTTTTATACTAAATTATTATGAAAATAATTGAATAATTAACTTTCAAACTAATAATTTTTTAATAAACYTATTTTTAATYCTTTCTTTT

TTTTCTATTTTTGGTTTATTTTTAATTTCACTAGATATTGGAACATTATATTTTATTTTTGGAGCATGAGCCGGAATAGTAGGAACATCTCTAAGAATTT
TAATTCGAGCCGAACTTGGACACCCTGGAGCATTAATTGGTGATGATCAAATTTATAATGTAATTGTTACAGCTCACGCTTTTATTATGATTTTTTTCAT
AGTAATACCTATTATAATTGGAGGATTTGGAAATTGATTAGTACCTCTTATATTAGGGGCACCTGATATAGCTTTCCCTCGAATAAATAATATAAGTTTT
TGACTTCTTCCTCCTGCTTTATCTCTTTTATTAGTAAGAAGTATAGTTGAAAATGGAGCTGGAACAGGTTGAACAGTTTACCCACCTTTATCAGCAGGAA
TTGCTCATGGAGGAGCTTCAGTTGATCTGGCTATTTTCTCTTTACATTTAGCCGGAATTTCTTCAATTTTAGGGGCTGTAAATTTTATTACTACAGTAAT
TAATATACGATCTACAGGAATTACTCTTGATCGAATACCTTTATTTGTTTGATCAGTTGTAATTACAGCTTTATTATTATTATTATCTTTACCAGTTTTA
GCGGGAGCTATTACTATATTATTAACAGATCGAAATTTAAACACTTCTTTTTTTGACCCTGCTGGAGGAGGAGATCCTATTTTATACCAACATTTATTTT
GATTTTTTGGTCATCCAGAAGTTTATATTTTAATTTTACCTGGATTTGGAATAATTTCTCATATTATTAGTCAAGAATCAGGTAAAAAGGAAACATTTGG
GTCTTTAGGAATAATTTATGCTATACTAGCAATTGGCTTATTAGGATTTATTGTATGAGCTCATCATATATTTACTGTAGGAATAGACGTAGATACTCGA
GCTTACTTTACATCTGCAACTATAATTATTGCTGTTCCAACTGGAATTAAAATTTTTAGTTGATTAGCCACATTACATGGAACTCAATTATCTTATTCTC
CTGCAATTCTTTGAGCTTTAGGATTTGTATTTTTATTTACTGTAGGAGGACTAACTGGAGTAGTTTTAGCTAATTCTTCAGTAGATATTATTTTACATGA
TACTTATTATGTAGTTGCTCATTTTCACTATGTTTTATCAATAGGAGCTGTATTTGCTATTATAGCAGGATTTATCCATTGATATCCTTTATTTACAGGA
TTAACTTTAAACGCTAAATGATTAAAAAGTCAATTTATTATTATATTCATTGGAGTAAATTTAACATTTTTCCCTCAACATTTTTTAGGGTTAGCAGGAA
TACCTCGACGATATTCTGATTACCCAGATGCTTACACTACATGAAACATTGTATCTACAATTGGTTCTTCAATTTCATTATTGGGAATTTTATTTTTCTT
TTTCATTATTTGAGAAAGTTTAGTATCTCAACGACAAGTAATTTATCCAATTCAATTAAATTCTTCTATTGAATGATACCAAAATACTCCTCCTGCTGAA
CATAGTTATTTAATAGAACAATTAATTTTTTTTCATGATCATGCATTATTAATTTTAGTAATAATCACTGTTTTAGTAGGATATTTAATATTTATATTAT
TTTTTAATTCATATATTAATCGATTTTTATTACATGGACAATTAATTGAAATAATTTGAACTATTCTCCCAGCTATTATTTTATTATTTATTGCTCTTCC
TTCATTACGACTTTTATACCTTTTAGATGAAATTAATGAACCTTCTGTTACTTTAAAAAGTATTGGTCACCAATGATACTGAAGTTATGAATATTCAGAC
TTTAATAATATTGAATTTGATTCATATATAATCCCAACTAATGAATTAGCAACTGATGGATTTCGTCTTTTAGATGTTGATAACCGAGTAGTTTTACCAA
TAAATTCTCAAATTCGAATTTTAGTGACTGCTGCAGATGTAATTCATTCATGAACTATTCCTGCCTTAGGAGTAAAGGTTGATGGAACCCCAGGACGACT
TAATCNAACAAATTTTTTTATT

D_tripunctata
??
??
??
??
??
??
??
??
??
??
??
??
??
??
??
??
??
??
??
??
??
??
??
??
??
??
??
??
??
??
??
??
??
??
??
??
??
??
?????????????????????????
AATTCTTCAAAAATTTTATTTTTAATAATAATAGTTATAGGAACAATAATTACAGTAACTTCTAATTCTTGATTAGGAGCTTGAATAGGACTTGAAATTA
ATTTGTTATCTTTTATCCCCCTAATAAGAGATAGTAATAATTTAAAATCAACTGAGTCTTCTTTAAAGTACTTTTTAACTCAAGCTTTAGCTTCTACTGT
TCTTTTATTTGCTGTAATTTTATTAATATTAAAAAATAATATAAATAACGAAATTAATGAATCTTTTACATCAATAATTTTAATATCAACATTACTGTTA
AAAAGAGGAGCTGCACCTTTTCATTTTTGGTTCCCAAATTTAATGGAAGGATTAACTTGAATAAATGCTTTACTTTTAATAACTTGACAAAAAATTGCCC
CTTTAATATTAATTTCTTATTTAAATTTAAATAATTTATTAATAATTAGAGTAATTTTATCAGTTATTGTTGGAGCATTA????????????????????
??
??
??
??
??
??????
GAACACTATATTTTATTTTTGGTGCTTGAGCCGGAATAGTAGGTACATCCCTAAGAATTTTAATTCGTGCTGAATTAGGACATCCTGGGGCATTAATTGG
AGATGATCAAATTTATAATGTAATTGTTACAGCTCATGCTTTTGTAATAATTTTTTTTATAGTAATACCAATCATAATTGGAGGGTTCGGAAATTGATTA
GTTCCTTTAATATTAGGAGCTCCCGATATAGCTTTCCCTCGAATAAATAATATAAGATTTTGACTACTCCCTCCTGCTCTAACATTATTATTAGTTAGAA
GTATGGTTGAAAACGGAGCTGGAACAGGTTGAACTGTTTACCCCCCATTATCAAGAGGTATTGCCCATGGAGGTGCCTCTGTTGATTTAGCAATTTTTTC
TCTTCATTTAGCTGGAATTTCCTCAATTTTAGGAGCAGTAAATTTTATTACAACCGTTATTAATATACGATCAACTGGTATTACTCTTGATCGAATACCC
TTATTTGTTTGATCAGTTGTAATTACAGCACTATTATTATTATTATCTTTACCAGTTTTAGCTGGAGCAATTACTATGCTTTTAACAGATCGAAATTTAA
ATACATCTTTCTTTGACCCAGCTGGGGGAGGGGATCCAATTTTATACCAACATTTATTTTGATTTTTTGGACACCCTGAAGTATACATTGTAATTAATCC

```
TGGATTTGGAATAATTTCTCATATTATTAGTCAAGAATCAGGTAAAAAGGAAACTTTTGGGTCTCTTGGAATAATTTATGCTATATTAGCTATTGGACTA
TTAGGGTTTATTGTATGAGCCCACCATATATTTACTGTTGGAATAGACGTTGATACGCGAGCCTATTTTACATCAGCTACAATAATTATTGCGGTTCCAA
CAGGAATTAAAATTTTCAGATGATTAGCTACTCTTCACGGAGCACAGTTATCTTATTCACCAGCTATTTTATGAGCTTTAGGATTTGTATTTTTATTCAC
AGTTGGAGGATTAACAGGAGTTGTTCTTGCAAATTCATCTGTTGATATTATTTTACATGATACTTATTATGTTGTTGCCCACTTTCATTATGTTCTCTCA
ATAGGAGCTGTATTTGCTATTATAGCTGGATTTGTACACTGATACCCTTTATTTACTGGACTAACATTAAATAATAAGTGATTAAAAAGTCAATTTATTA
TTATATTTATTGGAGTAAATTTAACATTTTTCCCTCAACACTTTCTTGGACTAGCTGGTATGCCTCGTCGTTATTCTGATTACCCTGACGCTTATACTTC
ATGAAATGTAATTTCAACTATTGGTTCTTCAATTTCTCTTTTAGGAATTTTATTCTTTTTTTATATTATCTGAGAAAGTTTAATTTCTCAACGACAAGTA
ATTTATCCTGTACAACTAAATTCATCAATTGAATGATATCAAAATACTCC????????????????????
TAATAGAACAATTAATTTTTTTTCATGATCATGCCTTATTAATTTTAGTTATAATTACTGTATTAGTAGGATATTTAATATTTATATTATTTTTTAATAG
ATATATTAATCGATTTTTATTACATGGTCAATTAATTGAAATAATTTGAACAATTTTACCTGCTATTATTTTATTATTTATTGCTCTCCCTTCTCTTCGC
TTATTATACTTATTAGATGAAATTAATGAACCTTCAGTAACTTTAAAAAGAATTGGGCATCAATGATACTGAAGCTATGAATATTCTGATTTTAATAATG
TTGAATTTGATTCTTATATAATTCCAACAAATGAGTTAGAAAATGACGGATTTCGACTTCTTGACGTTGATAATCGAATTGTACTCCCTATAAATTCCCA
AATTCGAATTTTAGTAACTGCCGCTGATGTAATTCATTCATGAACAATCCCTGCTTTAGGTGTAAAGGTTGATGGAACTCCAGGTCGTTTAAATCAAACT
AATTTTTTTATA

;
end;

begin assumptions;
	charset amy = 1-855;
	charset amyrel = 856-1332;
	charset boss = 1333-1698;
	charset fkh = 1699-2160;
	charset marf = 2161-2553;
	charset sina = 2554-2949;
	charset snf = 2950-3228;
	charset wee = 3229-3822;
	charset mito = 3823-6813;

end;
```
